# Role of CSF1R 550th-tryptophan in kusunokinin and CSF1R inhibitor binding and ligand-induced structural effect

**DOI:** 10.1038/s41598-024-63505-x

**Published:** 2024-05-31

**Authors:** Chompunud Chompunud Na Ayudhya, Potchanapond Graidist, Varomyalin Tipmanee

**Affiliations:** 1 Department of Biomedical Sciences and Biomedical Engineering, Faculty of Medicine, Prince of Songkla University, Hat Yai, 90100 Songkhla Thailand; 2 Bioactivity Testing Center, Faculty of Medicine, Prince of Songkla University, Hat Yai, 90100 Songkhla Thailand

**Keywords:** Biophysics, Computational biology and bioinformatics, Drug discovery

## Abstract

Binding affinity is an important factor in drug design to improve drug-target selectivity and specificity. In this study, in silico techniques based on molecular docking followed by molecular dynamics (MD) simulations were utilized to identify the key residue(s) for CSF1R binding affinity among 14 pan-tyrosine kinase inhibitors and 15 CSF1R-specific inhibitors. We found tryptophan at position 550 (W550) on the CSF1R binding site interacted with the inhibitors' aromatic ring in a π–π way that made the ligands better at binding. Upon W550-Alanine substitution (W550A), the binding affinity of *trans*-(−)-kusunokinin and imatinib to CSF1R was significantly decreased. However, in terms of structural features, W550 did not significantly affect overall CSF1R structure, but provided destabilizing effect upon mutation. The W550A also did not either cause ligand to change its binding site or conformational changes due to ligand binding. As a result of our findings, the π–π interaction with W550's aromatic ring could be still the choice for increasing binding affinity to CSF1R. Nevertheless, our study showed that the increasing binding to W550 of the design ligand may not ensure CSF1R specificity and inhibition since W550-ligand bound state did not induce significantly conformational change into inactive state.

## Introduction

Tyrosine kinases (TKs) regulate the signal transductions which control a wide range of fundamental cellular functions, including cell growth and proliferation, differentiation, migration, metabolism, and mortality^1,2^. At least 90 unique TK genes have been identified. In these numbers, 58 of them encode receptor tyrosine kinases (RTKs)^3^. Dysregulation of RTK signaling impacts various diseases, especially cancer^4^, as they promote tumorigenesis^5^, migration^6^, as well as tumor angiogenesis^7^. Therefore, RTKs have emerged as therapeutic targets and clinical prognostic factors for cancer^8–10^.

Among 20 RTK subfamilies, RTK class III (PDGFR family) which includes PDGFRα, PDGFRβ, C-KIT, CSF1R, and FLT3, is highly expressed in various types of epithelial cancers^9^ and associated with poor prognosis^11,12^ via promoting tumor angiogenesis and metastasis^1,6,12^. The Colony stimulating factor-1 receptor (CSF1R or FMS) is RTK class III that regulates immune responses^13,14^ and reproductive functions, including mammary gland development^15^ and regulation of ovulation rates^16^. The expression of CSF1R and its native ligand CSF-1 strongly correlates with oncogenesis and poor prognosis of breast carcinomas among other epithelial tumors^17–19^. Activation of CSF1R by CSF-1 triggers phosphorylation cascades such as PI3K-AKT, MAPK and STAT pathway^20,21^ which promote breast cancer cell proliferation^22^, metastasis^23^, and angiogenesis^19^, leading to the enhanced invasiveness of breast cancer^17–19,24^.

CSF1R-derived PI3K-AKT pathway induces c-*myc* gene expression, leads to upregulation of cyclinB, cyclinD, cyclinA and CDKs which drives cell cycle resulting in cancer cell proliferation and survival^25^. The MAPK pathway activates RAS-RAF-MEK-ERK, consequently phosphorylates transcription factors including RUNX which regulates expression of *csf1r* gene^26^. The expression of *csf1r* gene, which produces CSF1R and CSF1, is strongly associated with poor outcome in breast cancer and results in tumor cell invasiveness and pro-metastatic behavior both in patient and in vitro^24,27–32^. CSF1R activation also triggers the STAT1/3/5 pathways, which regulates several target oncogenes and affects cancer progression, proliferation, anti-apoptosis, metastasis, as well as chemoresistance and survival activities^21,33,34^.

Activation of CSF1R also enhances recruitment of tumor-associated macrophages (TAMs) to the tumor site, hence the poor prognosis was observed^23,35,36^. The high expression levels of CSF1R and its ligand, CSF-1, have been found to correlate with poor prognosis in many cancer types such as breast carcinoma^17–19,24,37^, ovarian carcinoma^38^, lung adenocarcinoma^39^, gastric adenocarcinoma^40^, prostate adenocarcinoma^41^ and leukemia^42^. With PLX3397 (pexidartinib) been approved by the FDA for the treatment of tenosynovial giant cell tumor in 2019^43^, pharmacological inhibition of CSF1R has emerged as a promising antitumor strategy. Therefore, targeting CSF1R with specific inhibitors has become an attractive therapeutic option and several CSF1R inhibitors are currently developed^10,44,45^.

The first pan-tyrosine kinase inhibitor (TKI) imatinib was approved by the FDA in 2001^46^. It is commonly used as an RTK class III pan-inhibitor^47^. However, prolonged imatinib treatment may cause drug resistance via mutation in cancer cells^48^ as well as adverse effects due to its ability to also target other kinases^49,50^. Henceforth several single or dual-specific RTK class III inhibitors were developed, such as pexidartinib which was approved as CSF1R specific inhibitor by the FDA in 2019^43^. However, one of the biggest challenges of designing a specific drug for RTKs is that the RTKs share similar protein structures^1,3,8,51,52^. Therefore, searching for new, lower toxic and higher target-specificity, TKIs is still of interest.

According to in silico molecular docking studies^53,54^ and in vitro si-RNA studies^53^, *trans*-(−)-kusunokinin, a lignan compound found in *Piper nigrum*^35^, targeted CSF1R, inhibiting the receptor and AKT signaling cascade, thus suppressing cancer cell proliferation and metastasis. *Trans*-(−)-kusunokinin inhibits cancer cell growth, proliferation and metastasis in a variety of cancer cell lines, including breast, colon and lung cancer cell lines^55^. The compound also reduces the size of breast tumors in mouse models without affecting body weight, internal organs, or bone marrow^56^. The synthetic racemic mixture *trans*-(±)-kusunokinin also showed anticancer activities against breast, colon, cholangiocarcinoma^57^ and ovarian cancer cell lines^58^ with low toxicity against normal cells^59^. The *trans*-(−)-kusunokinin was hypothesized to be the main active component in the racemic mixture, targeting proteins in proliferation and metastasis pathway, while *trans*-(+)-kusunokinin may additionally inhibit other related molecules. The differences in binding modes between the kusunokinin isoforms to CSF1R were noticed and the stereoselectivity of CSF1R for *trans*-(−)-kusunokinin over *trans*-(+)-kusunokinin was previously proposed^54^.

Since the ligand-induced conformational effect on the CSF1R could be the key understanding how the inhibitor triggered the CSF1R inhibition, however, the information about how inhibitor affected the CSF1R structurally remain unknown. In this study, we utilized in silico methods to visualize the significance of CSF1R-inhibitor interacting residues by performing molecular docking to compare the interacted residues and CSF1R specificity of pan-TKIs and CSF1R-specific inhibitors. In silico Alanine-mutagenesis and Molecular Dynamics simulation (MD) were also performed to elucidate the impact of interacted residue(s) on the ligand-receptor structural-activity relationships.

## Results

### Molecular docking in comparison of pan-TKIs and CSF1R-specific inhibitors

Fourteen pan-TKIs and fifteen single- or dual-CSF1R specific inhibitors (CID were provided in Table S1) were docked to CSF1R kinase domain 4R7H (www.rcsb.org/structure/4R7H) which originally bound to pexidartinib, the first FDA-approved CSF1R-specific inhibitor, to compare the binding affinity of the ligands. Prior to this docking procedure, all tested ligands were docked to CSF1R kinase domain in different states (as listed in Table S1) to verify if they were type-II TKIs which preferably bound the protein in inactive state (DFG-out) to active state (DFG-in), to reduce the possible error due to the CSF1R 4R7H conformation is in inactive state. All docked ligands showed better or similar binding energies to CSF1R inactive state than CSF1R active state (Table S2) and were proceeded to docked to the CSF1R 4R7H.

The docking score from the best binding pose, shown in Table 1, represented the binding energy of the ligand in CSF1R binding pocket and was used to interpret the binding affinity. The lower binding energy indicated the higher possibility of good binding affinity of the ligand to the protein.

**Table 1:** Docking scores of CSF1R kinase 4R7H and tested ligands.

Docked ligand	Docking scores (kcal/mol)
Tested compounds (2 compounds)	
* Trans*-(−)-kusunokinin	− 11.47
* Trans*-(+)-kusunokinin	− 9.87
Pan-TKIs (14 compounds)	
Chiauranib	− 12.96
Pazopanib	− 11.95
Quizartinib	− 11.54
Sorafenib	− 11.46
Imatinib	− 11.16
Linifanib	− 10.99
Nilotinib	− 10.58
Sunitinib	− 10.11
Dasatinib	− 9.93
Tandutinib	− 9.67
OSI-930	− 9.48
Sulfatinib	− 9.15
Dovitinib	− 8.95
Tinengotinib	− 8.34
CSF1R-specific inhibitors (15 compounds)	
BPR1R024	− 11.67
Pexidartinib	− 11.07
Sotuletinib	− 10.55
KI20227	− 10.2
PLX5622	− 9.96
IACS-9439	− 9.90
JNJ28312141	− 9.52
JTE-952	− 9.51
Pimicotinib	− 9.45
Edicotinib	− 9.38
Vimseltinib	− 9.25
GW2580	− 8.87
ARRY-382	− 8.75
Q27456873	− 8.60
AZD7505	− 7.97

Both pan-TKIs and CSF1R-specific inhibitors, showed good docking scores when docked to CSF1R 4R7H, indicated that they all possibly bound CSF1R. No significant differences in binding affinity were found between the two groups. Therefore, docking score alone was not enough to determine CSF1R-specificity. However, the differences in docking scores between each docked ligand were noticed.

Chiauranib, the pan-TKI targeted various angiogenesis-related kinases including CSF1R^60^, showed the best binding energies (docking scores = − 12.96 kcal/mol), followed by pan-TKI pazopanib^61^ and CSF1R specific inhibitor BPR1R024^62^ (docking scores = − 11.95 and − 11.67 kcal/mol, respectively). Interestingly, BPR1R024 and several pan-TKIs showed better binding energy than pexidartinib which had docking score = − 11.07 kcal/mol. Our compound of interest: *trans*-(−)-kusunokinin also showed better binding energy than pexidartinib while *trans*-(+)-kusunokinin showed lesser binding affinity (docking scores = − 11.47 and − 9.87 kcal/mol, respectively).

Surprisingly, pan-TKIs bound CSF1R with overall binding affinity better than CSF1R-specific inhibitors. The average docking score within pan-TKIs group was − 10.4479 kcal/mol whereas the average docking score within CSF1R-specificinhibitors group was − 9.6433 kcal/mol. The average docking score between all groups was − 10.0871 kcal/mol.

Using the average docking score between all groups as a cut-off, 8 out of 14 pan-TKIs shower high binding affinity to CSF1R kinase. On the other hand, only 4 out of 15 specific CSF1R inhibitors pass the cut-off. *Trans*-(−)-kusunokinin showed higher binding affinity than the cut-off whereas *trans*-(+)-kusunokinin binding affinity, in contrast, was below the cut-off. Interaction analysis was performed to identify the binding residue(s) that influenced the ligand binding affinity.

### Interacted residue frequency revealed key residue for good binding affinity

Binding pose of each docked ligand with the lowest binding energy and the same binding site to pexidartinib were consequently subjected to interaction analysis. Interacted residues were listed in Table S3. Most of the docked ligands were found to form H-bond with Cysteine position 666th which is the ATP-binding residue^63^ (20 out of 31 ligands), followed by Glutamic acid position 664th and Asparagine position 796th of the DFG motif^44^ (11 out of 31 each). Some other frequently found interacted residues were Glutamic acid position 633rd (formed H-bound with 8 out of 31 docked ligands), Arginine position 549th, Leucine position 588th and Asparagine position 670th (each residue formed H-bound with 5 out of 31 docked ligands).

Six docked ligands formed either π–π or π–T shape with Tyrosine position 665th (Y665). However, among these 6 ligands, only imatinib which formed π–π interaction with Y665 in addition to π–π stacking with W550 and π–T shape with F797 had binding affinity above the cut-off (Table S3).

It was found that Phenylalanine position 797th (F797) of the conserved DFG-motif^44^ formed either π–π or π–T shape with 16 out of 31 ligands (Table 2) but with no notable correlation with the ligand binding energy (6 ligands were above the cut-off, 10 ligands were below the cut-off). So, it was though that although F797 was a mandatory interacted residue for ligand binding in CSF1R pocket, it had no impact on CSF1R binding affinity.

**Table 2:** Interacted residues frequency within each docked ligand groups.

Docked ligand group/tested ligand	Number of ligands which form π–π interaction with W550		Number of ligands which form π–π interaction with F797	
	Docking score above cut-off	Docking score below cut-off	Docking score above cut-off	Docking score below cut-off
Pan-TKIs (14 compounds)	5	1	5	2
CSF1R-specific inhibitors (15 compounds)	4	0	1	7
*Trans*-(−)-kusunokinin	1	–	–	–
*Trans*-( +)-kusunokinin	–	–	–	1
Total number	10	1	6	10

**Figure 1:**
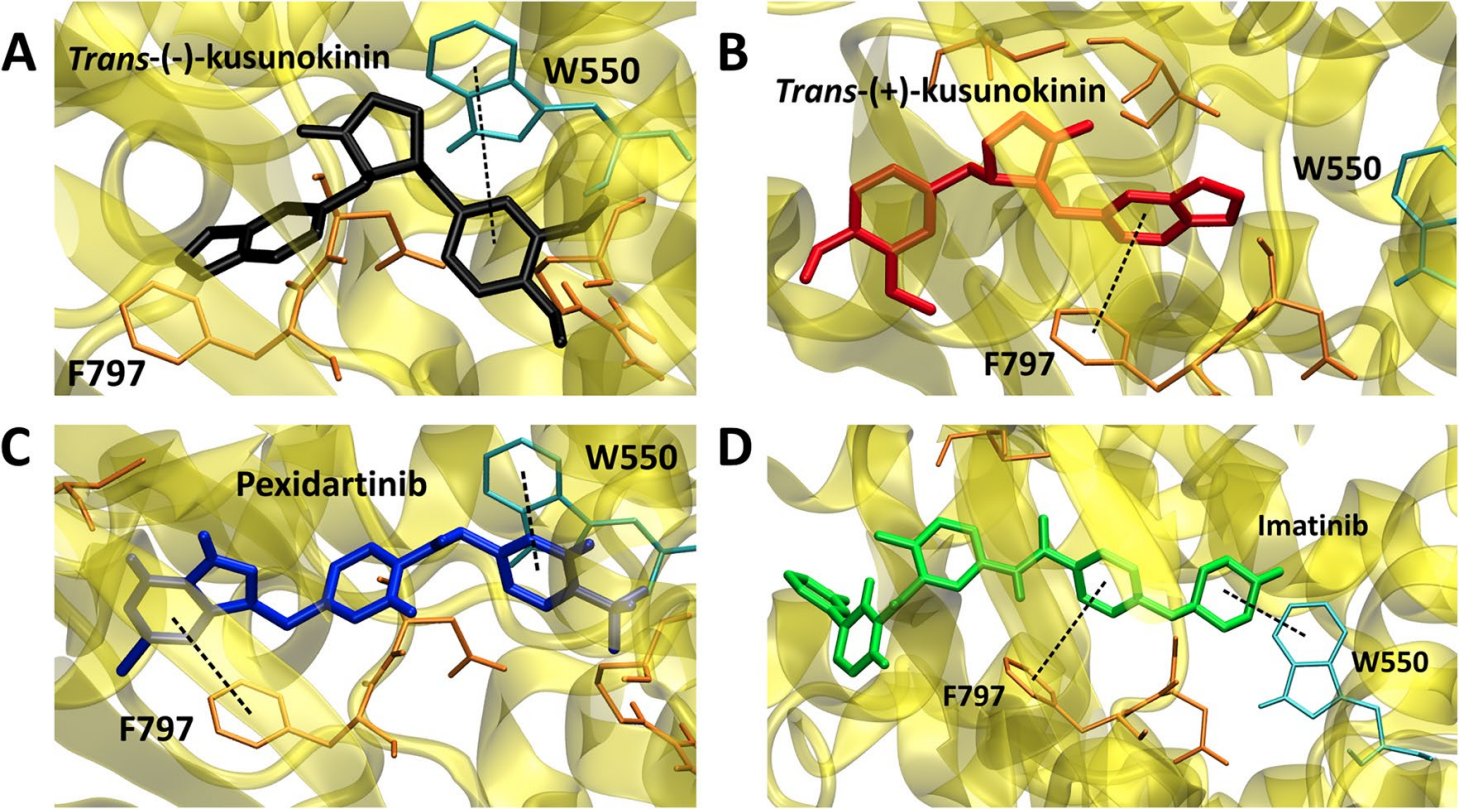
π–π stacking from the bound ligand with W550 in CSF1R^WT^. (**A**) *Trans*-(−)-kusunokinin (black), (**B**) *Trans*-(+)-kusunokinin (red), (**C**) Pexidartinib (blue), and (**D**) Imatinib (green) were depicted and the dash line indicated π–π interaction between the amino acid and the ligand. The protein and ligand structures were generated using Visual Molecular Dynamics (VMD) software version 1.9.3

On the contrary, Tryptophan position 550th (W550) of the CSF1R juxtamembrane domain^44,63^ was noticed to form π–π stacking with 11 out of 31 docked ligands (Table 2). In these numbers, 10 of them were above the cut-off. It was even more obvious in CSF1R specific inhibitors group which all the 4 specific CSF1R inhibitors that pass the cut-off formed π–π stacking with W550 while none of the 11 ligands below the cut-off interacted with W550. *Trans*-(−)-kusunokinin which showed better binding energy than *trans*-(+)-kusunokinin also bound CSF1R at W550 while *trans*-(+)-kusunokinin bound CSF1R at F797. Figure 1 depicted the π–π stacking from the bound ligand with W550. Therefore, W550 was chosen to perform Alanine mutagenesis to validate the impact of this residue.

### Alanine mutagenesis

To investigate the significance of interaction at CSF1R W550, W550 was replaced with Alanine (CSF1R^W550A^), which omits the aromatic side chain while retaining the main-chain conformation and charges. Following the introduction of mutations, the side chain and protein folding were optimized. Structural changes upon mutation were examined and found that the mutation led to destabilizing effects^64^ on the protein (ΔΔG =  + 2.23696 kcal/mol).

### Molecular dynamics (MD) simulations analysis

Although molecular docking is a useful tool to investigate ligand–protein binding characters^65,66^, it cannot predict protein conformational changes upon ligand binding or kinetics due to docking procedures performed on a rigid protein model. Therefore, MD simulations were performed to evaluate the binding interaction that would occur in nature^67–72^. Widely used pan-TKIs: imatinib^45,46^ was chosen as a representation of pan-TKIs group while pexidartinib represented specific CSF1R inhibitor^43^, compared to our compounds of investigation: *trans*-(−)-kusunokinin and *trans*-(+)-kusunokinin. MD trajectories were obtained and proceeded to post-MD analysis.

#### Root mean square deviation (RMSD) and root mean square fluctuation (RMSF)

**Figure 2:**
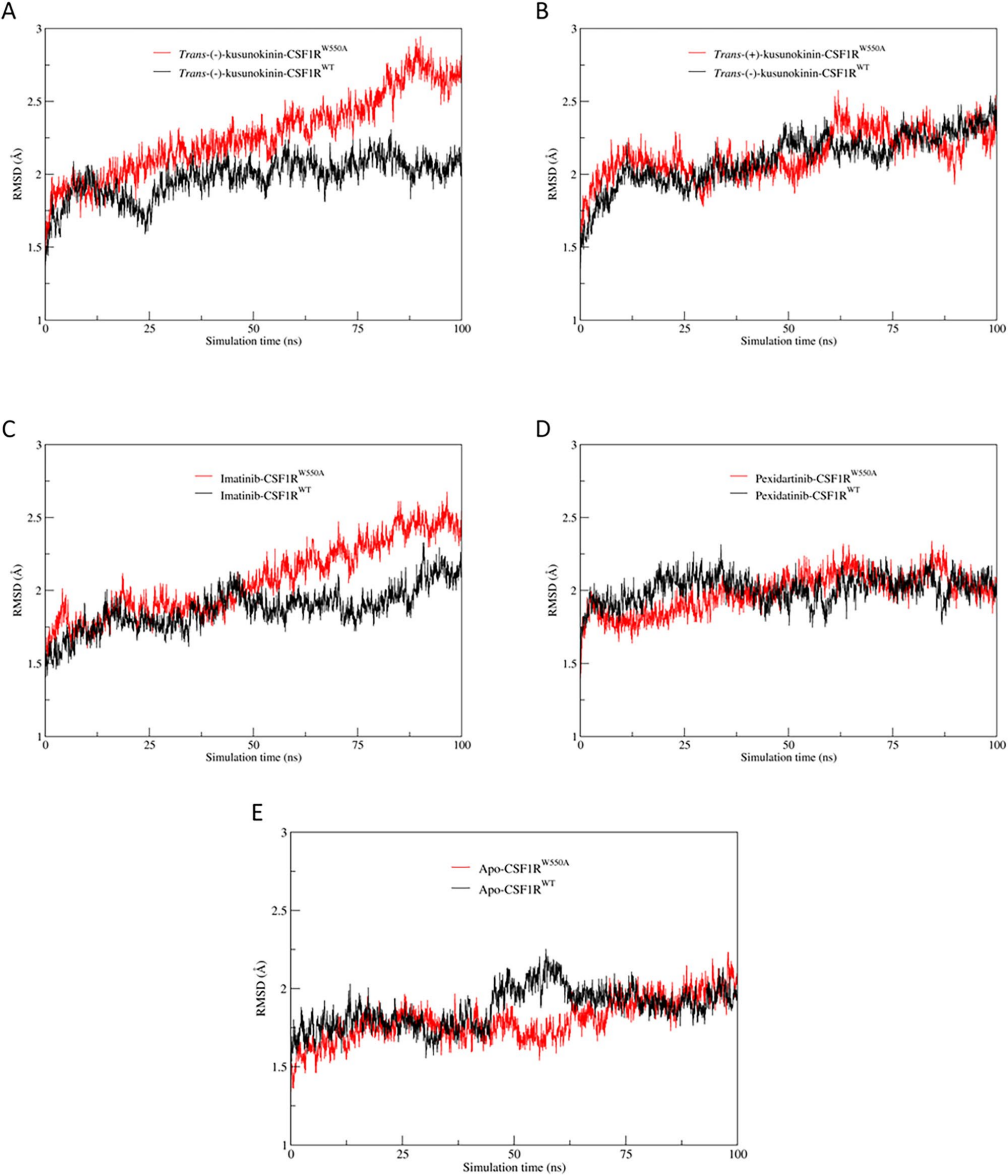
Root-mean square distance (RMSD) plot of the protein backbone. (**A**) *Trans*-(−)-kusunokinin in complex with CSF1R^W550A^ (red) or CSF1R^WT^ (black). (**B**) *Trans*-(+)-kusunokinin in complex with CSF1R^W550A^ (red) or CSF1R^WT^ (black). (**C**) Pexidartinib in complex with CSF1R^W550A^ (red) or CSF1R^WT^ (black). (**D**) Imatinib in complex with CSF1R^W550A^ (red) or CSF1R^WT^ (black). (**E**) Ligand-free CSF1R^W550A^ (red) or CSF1R^WT^ (black). The values were calculated from three independent simulation systems and represented as mean values.

First, RMSD was used to ensure that the complexes remained stable throughout the simulations. The RMSD analysis, illustrated in Fig. 2, revealed that every tested ligand remained in the pocket consistently throughout the simulations.

It was found from Fig. 2A and C that mutation W550A leaded to instability of the *trans*-(−)-kusunokinin-CSF1R^W550A^ complex after 50 ns of simulations and imatinib-CSF1R^W550A^ complex over the last 25 ns of simulation time, compared to CSF1R^WT^ (Fig. 2E). *Trans*-(+)-kusunokinin also showed subtler climb-up fluctuations in the backbone RMSD after 50 ns of simulations in both models (Fig. 2B). However, the values are still within an acceptable range to be deemed stable enough to determine conformational changes upon ligand binding (RMSD larger than 3.0 Å)^65^. Pexidartinib, on the other hand, dissipated stable binding complexes throughout the simulation both in wild-type and mutant models (Fig. 2D).

Apart from the binding stability via the view of RMSD and ligand distance, fluctuations of protein residues upon ligand binding were evaluated with the RMSF of the α-carbon in each amino acid residue to determine the stability of the system as well as indicate the interacting residues that ligands are bound to. The original and re-arranged residue numbers were paired as shown in Table S4.

**Figure 3:**
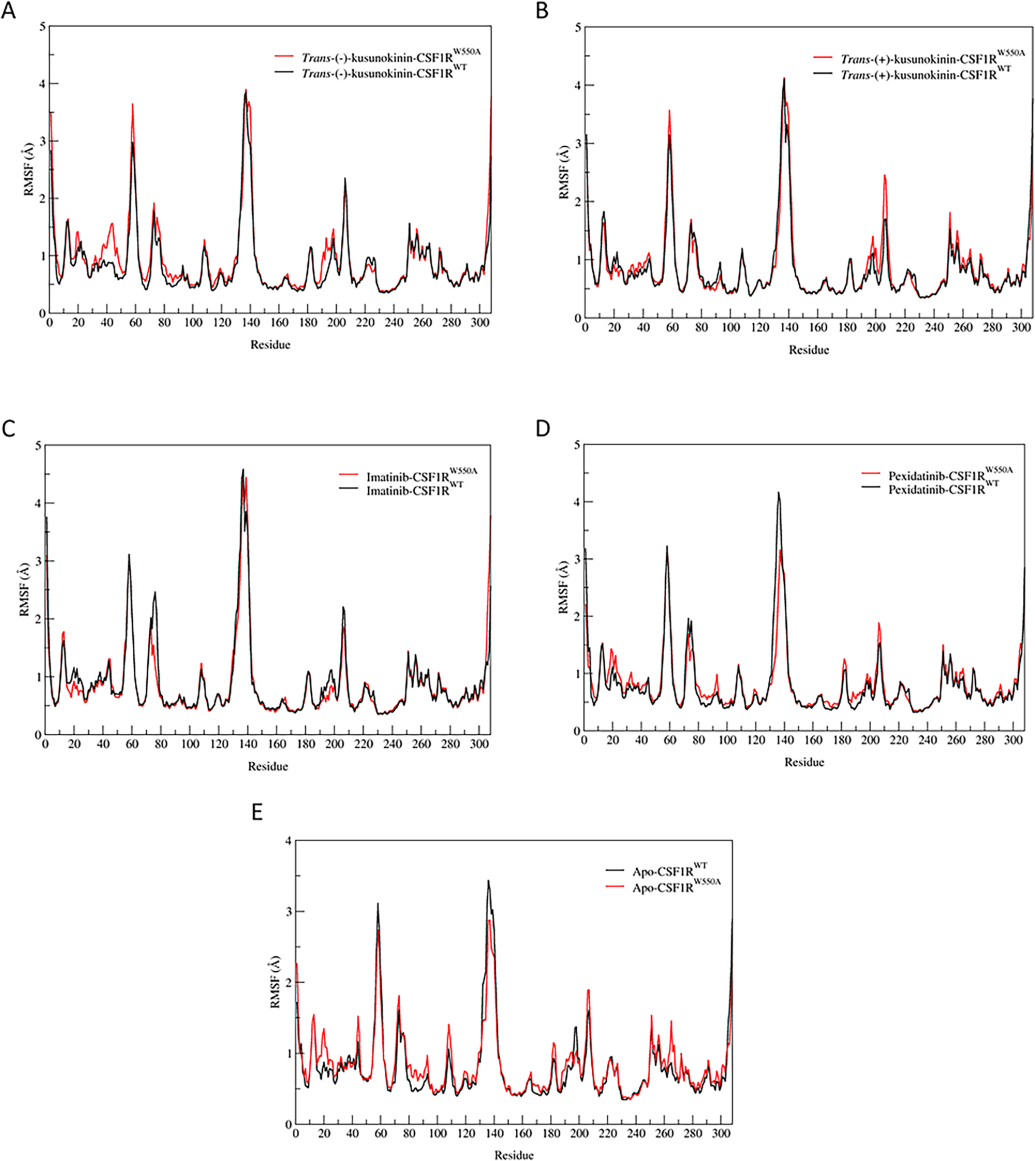
Root-mean square fluctuation (RMSF) plot of the α-carbon position in the protein backbone. (**A**) *Trans*-(−)-kusunokinin in complex with CSF1R^W550A^ (red) or CSF1R^WT^ (black). (**B**) *Trans*-(+)-kusunokinin in complex with CSF1R^W550A^ (red) or CSF1R^WT^ (black). (**C**) Pexidartinib in complex with CSF1R^W550A^ (red) or CSF1R^WT^ (black). (**D**) Imatinib in complex with CSF1R^W550A^ (red) or CSF1R^WT^ (black). (**E**) Ligand-free CSF1R^W550A^ (red) or CSF1R^WT^ (black). The values were calculated from three independent simulation systems and represented as mean values. Residue numbers were re-arranged according to AMBER procedure.

The RMSF patterns of CSF1R^WT^ and CSF1R^W550A^ were similar for all tested ligands (Fig. 3). This implied that the ligand binding could contribute no significant effect on the CSF1R structure flexibility, in all WT, mutant and ligand-free forms.

#### CSF1R conformational change upon alanine mutagenesis

**Figure 4:**
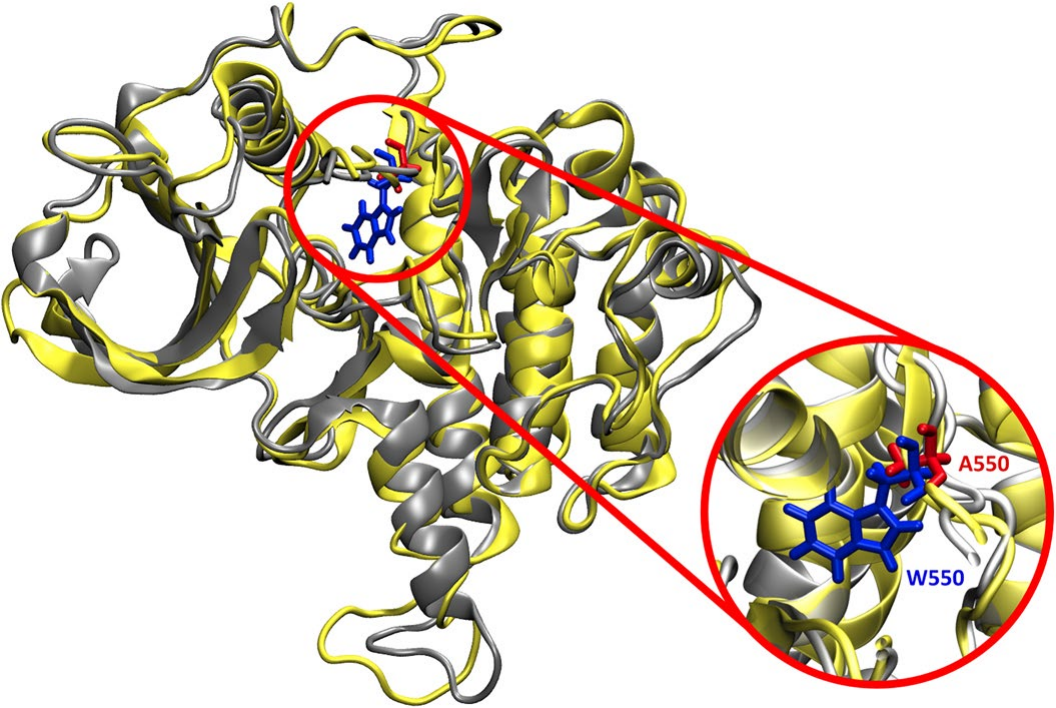
Alignment of ligand-free CSF1R kinase structure. Yellow structure represented CSF1R^WT^ and grey structure represented CSF1R^W550A^. Blue sticks represented the native residue W550 of CSF1R^WT^ and red sticks represented the mutated residue A550 of CSF1R^W550A^. Circle highlighted the substituted site. The protein structures were generated using Visual Molecular Dynamics (VMD) software version 1.9.3

**Figure 5:**
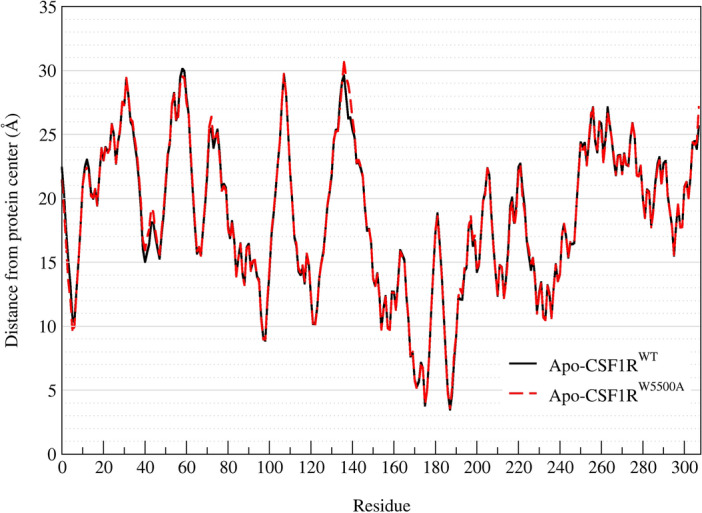
Average distance from CSF1R kinase center to each residue backbone, calculated from the last 800 MD snapshots from the triplicated MD systems. Similar distance patterns suggested no conformational changes between the two models.

Although the mutation W550A led to destabilizing effects on the protein (ΔΔG =  + 2.23696 kcal/mol), calculated with FoldX command, it did not change the protein conformation. The conformation alignment of ligand-free CSF1R^WT^ and CSF1R^W550A^ MD simulations in Fig. 4 showed to be resemble. The average distance pattern from protein center to the backbone of each residue also indicate no significant conformational changes (Fig. 5).

#### Distances between center of ligand and center of protein during simulations

**Figure 6:**
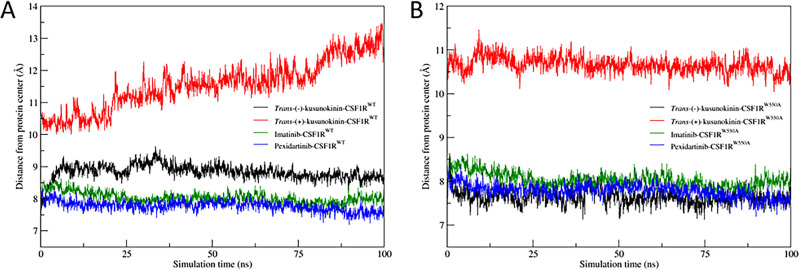
Distances between center of ligand and center of protein during simulations. (**A**) Ligand-CSF1R^WT^ models and (**B**) Ligand-CSF1R^W550A^ models. Distances between *trans*-(−)-kusunokinin to protein models were represented in black, *trans*-(+)-kusunokinin in red, imatinib in green and pexidartinib in blue. The values were calculated from three independent simulation systems and represented as mean values.

The distance between the ligand center of mass and the protein center of mass was plotted along the simulation to visualize the ligand's binding characteristics towards the protein. This could be investigated to see if the W550A mutation could repel the ligand or change its position in the CSF1R inhibitor binding pocket (Fig. 6).

The constant distance of *trans*-(−)-kusunokinin into the protein center, as well as imatinib and pexidartinib in both WT (Fig. 6A) and mutant CSF1R forms (Fig. 6B), suggested that all these compounds could remain in the CSF1R pocket. As a result, the W550A mutation may not have as strong an effect on the remnant drug characteristic binding in the CSF1R pocket as previously thought^34^. In other words, the W550A mutation had no effect on ligand locking in CSF1R. The greater distance of *trans*-(+)-kusunokinin to the protein center in both WT and mutant compared to other ligands, on the other hand, supported a previous study^54^ that the *trans*-(+)-kusunokinin may not preferentially bind the CSF1R-inhibitor site.

#### CSF1R conformational change upon ligand binding

**Figure 7:**
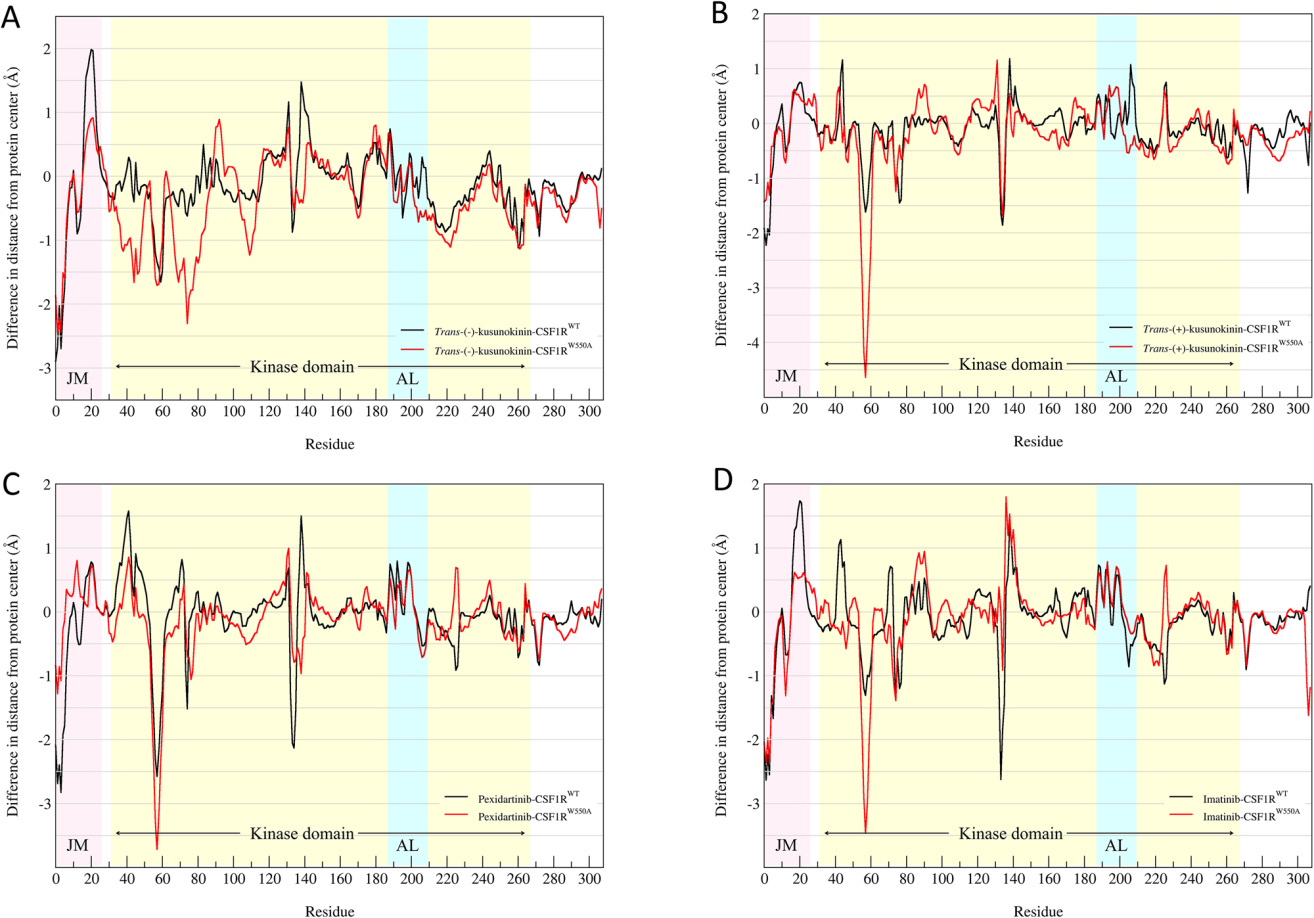
Difference in distance patterns upon ligand-binding between ligand-bound CSF1R^WT^ and ligand-bound CSF1R^W550A^, calculated from the ligand-free models. (**A**) *Trans*-(−)-kusunokinin in complex with CSF1R^W550A^ (red) or CSF1R^WT^ (black). (**B**) *Trans*-(+)-kusunokinin in complex with CSF1R^W550A^ (red) or CSF1R^WT^ (black). (**C**) Pexidartinib in complex with CSF1R^W550A^ (red) or CSF1R^WT^ (black). (**D**) Imatinib in complex with CSF1R^W550A^ (red) or CSF1R^WT^ (black). The light blue highlighted the activation loop (AL) region. The zero line represented the position of each residue from ligand-free CSF1R models. Minus values indicated that residues moved closer to the protein center while plus values indicated that residues moved further from the protein center. Similar distance patterns suggested no conformational changes between the two models. Differences of distance pattern more than 2.0 Å were counted as significant conformation changes due to mutation W550A.

Distance patterns were analyzed to see conformational changes in terms of movement of residue α-carbon from their original binding-free position to ligand-bound position. Difference in distance pattern between WT and W550A was plotted compared to original binding-free position (Fig. 7). Differences in distance pattern between WT and W550A more than 1.5 Å were counted as a slight conformational change upon ligand-binding due to W550A mutagenesis and more than 2.0 Å were counted as significant conformation changes (Table S5). These cut-offs were based on the distance which amino acid possibly form or loss their original interaction (interacted residue distance range from H-bond at 2 Å to hydrophobic interaction at 4 Å)^73^.

Interestingly, *trans*-(−)-kusunokinin showed only slight W550A mutagenesis impacted on residue number 70–78 (Fig. 7A). These residues were move closer to the center of protein mass, however, none of these residues were significantly affected by the mutation W550A. On the other hand, W550A significantly impacted amino acid position 603rd–609th (residue 55–61), especially Lysine position 606th (residue 58) in binding to *trans*-(+)-kusunokinin (Fig. 7B). Imatinib and pexidartinib also showed close to none W550A mutagenesis impacted on CSF1R conformational change upon ligand-binding.

#### DFG-in and DFG-out conformation analysis

X-ray crystal structures reveal remarkable conformational heterogeneity, with active (on state) and inactive (off state) conformations. When the protein is active, the aspartate of the DFG motif engages the ATP-binding site and coordinates two Mg^2+^ ions. The activation loop has an open and extended shape^63,74^. An active state conformation is also distinguished by the orientation of the C helix, which is located on the N-terminal domain^44^. In an active conformation, it rotates inward toward the active site, followed by a characteristic ion-pair interaction between the conserved Glu of the C helix and the Lys of the N lobe's strand^74–76^. Imatinib binds to the new allosteric pocket in this DFG-out conformation, sparking a great deal of interest in developing inhibitors that specifically target the inactive DFG-out conformation^77,78^.

Inhibition of CSF1R was indicated by the position of DFG-motif which move Asparagine position 796th (D796) away from ATP-binding site, lead to DFG-out state or inactive state due to catalytic incomplete thus open an allosteric pocket adjacent to the ATP-binding pocket, which usually is the binding pocket of type II TKIs^63^. The α-carbon distance between Asparagine position 783rd (D783) following the HRDxxxN motif and Phenylalanine position 797th (F797) of the DFG motif were represented as *d*_1_. The α-carbon distance between the conserved Glutamic acid position 633rd (E633) on the αC-helix and F797 of the DFG motif were represented as *d*_2_.

These distances were used to interpret CSF1R state which considered DFG-out or inactive state when *d*_1_ < *d*_2_ and considered DFG-in or active state when *d*_1_ > *d*_2_. The classical DFG-out conformation cut-off at *d*_1_ < 7.2 Å and *d*_2_ > 9 Å^61,63^. The average distance *d*_1_ and *d*_2_ of each ligand–protein systems were averaged from the last 800 snapshots from the triplicated MD simulations and summarized in Table 3. The *d*_1_ and *d*_2_ of each MD simulations, as well as the difference in *d*_1_ and *d*_2_ upon mutation W500A, were provided in Table S6.

**Table 3:** Conformational change in terms of DFG-state.

Ligand–protein	CSF1R^WT^		CSF1R^W550A^	
	*d*_1_	*d*_2_	*d*_1_	*d*_2_
*Trans*-(−)-kusunokinin	7.25 ± 0.14	10.35 ± 0.44	7.67 ± 0.13	10.94 ± 0.42
*Trans*-(+)-kusunokinin	7.37 ± 0.16	11.01 ± 0.27	7.62 ± 0.22	10.01 ± 0.32
Pexidartinib	7.16 ± 0.31	10.96 ± 0.48	7.52 ± 0.17	10.27 ± 0.50
Imatinib	7.07 ± 0.14	10.55 ± 0.07	7.39 ± 0.02	10.15 ± 0.38
Ligand-free	7.21 ± 0.34	11.30 ± 0.25	7.23 ± 0.03	11.97 ± 0.31

All tested ligand showed binding and preserving CSF1R in inactive DFG-out conformation without significant mutagenesis impact. The two reference ligands: imatinib and pexidartinib bound CSF1R in the classical DFG-out state while *trans*-(−)-kusunokinin and *trans*-(+)-kusunokinin showed slightly greater *d*_1_than the cut-off, indicated that these two compounds bound CSF1R in alternative (nonclassical) DFG-out inactive conformations. Moreover, *trans*-(+)-kusunokinin showed distance of *d*_2_ > 10.5 Å, therefore the binding conformation should be considered as αC-out conformation^62^, emphasized that *trans*-(+)-kusunokinin had different binding mode than *trans*-(−)-kusunokinin.

#### Relative binding free energies

Even though the structure parameter informed the binding characteristics of all studied ligands, the affinity had to be evaluated as the different binding site cannot assume the less binding affinity. The Molecular Mechanics/Generalized Born Surface Area (MM/GBSA)^79,80^ was thus used to calculate the ligand–protein complex's relative binding free energy. The free binding energies indicate how likely the ligand is to fit into the protein's binding pocket. Each system's MM/GBSA energies were calculated using three independent simulations and represented as mean standard error of mean (SEM). The statistical significance of binding free energy changes upon mutation was determined using the student's t-test (*p*-value < 0.05). All energies were reported in Table 4.

**Table 4:** MM/GBSA energies in kcal/mol.

Ligand–protein	CSF1R^WT^	CSF1R^W550A^	*p*-value
*Trans*-(−)-kusunokinin	− 50.85 ± 0.15	− 43.42 ± 0.14	< 0.0001
*Trans*-(+)-kusunokinin	− 33.92 ± 0.17	− 42.86 ± 0.15	< 0.0001
Imatinib	− 55.36 ± 0.22	− 68.14 ± 0.15	< 0.0001
Pexidartinib	− 62.27 ± 0.14	− 60.91 ± 0.14	0.0023

The substitution of W550 for Alanine did, in fact, have a significant effect on ligand binding to CSF1R. The mutation W550A reduced *trans*-(−)-kusunokinin and pexidartinib binding affinity to the CSF1R pocket while increasing *trans*-(+)-kusunokinin and imatinib binding. However, in both systems, the higher binding ability of *trans*-(+)-kusunokinin after mutation did not exceed *trans*-(−)-kusunokinin binding to the protein.

#### Off-target effects of trans-kusunokinin

Both *trans*-(−)-kusunokinin and *trans*-(+)-kusunokinin were analyzed for off-target predictions using Protox 3.0 for cytotoxicity (https://tox.charite.de/protox3/), SwissADME for drug-likeness (http://www.swissadme.ch/), and SwissTargetPrediction for drug-target prediction (http://www.swisstargetprediction.ch/). The off-target effects of these compounds are summarized in Figs. S1, S2, S3 and S4, respectively. In conclusion, both compounds exhibited the expected low toxicity and acceptable drug-likeness. The findings indicated that both *trans*-(−)-kusunokinin and *trans*-(+)-kusunokinin could be promising for the next stage of development.

## Discussion

### CSF1R-inhibitor interactions and modes of action

Receptor tyrosine kinases (RTKs) are crucial in regulating normal cellular function^1,2^. Numerous evidence linked malfunction of RTKs to various diseases, especially cancer^4^. The Colony stimulating factor-1 receptor (CSF1R or FMS) is RTK class III which strongly correlates with oncogenesis and poor prognosis of many types of cancer, including breast carcinomas^17–19^. Upon binding of its ligands: CSF-1 or IL-34, dimerization and activation of CSF1R consequently catalyzes the transfer of ATP phosphate to its tyrosine residue^44,63^, thus triggers breast cancer cell proliferation^22,25^, metastasis^23,31^, and angiogenesis^19^ pathways.

Since the first pan-tyrosine kinase inhibitor (TKI) imatinib was approved by the FDA in 2001^46^. TKIs with increased specificity and selectivity toward either single RTK target or multiple RTK targets have become a major area of anticancer drug discovery in the past 20 years^8,10^. CSF1R inhibitors are also rapidly developed^10,44,45^. Among numerous lead compounds, pexidartinib is the first small-molecule inhibitor targeting CSF1R specifically that is approved by the FDA in 2019^43^.

In this study, 14 pan-TKIs and 15 single or dual CSF1R-specific inhibitors were sampling to perform Molecular docking with CSF1R kinase domain crystalized structure to see if there are any correlation between the binding energy, which interpreted from the docking score, and the specificity of the ligand. As expected, although Molecular docking is a useful tool in drug discovery process, one of its limitations is that the calculated docking score from the rigid ligand and rigid protein docking is not necessarily relevant to the specificity or selectivity of the ligand tested in living organisms^66^. Therefore, it is not surprising to see that some of the pan-TKIs had better docking scores than CSF1R-specific inhibitors tested in this study.

We found that CSF1R’s Tryptophan position 550th is conserved across most species^82^ and various CSF1R inhibitors were designed based on the π–π interaction with the receptor, with W550 being the common interacting residue^54,83–87^. Based on this information, we have tried to elucidate and investigate the effect of the W550 and mutated ones on the drug-CSF1R interaction, as well as the effect on the structure of CSF1R. Due to this lack of these information, our study could provide more insight for the drug design guide based on W550, not only the binding enhancement but also the structural information of ligand bound CSF1R. However, the binding pose and interaction analysis gave us information on how to improve ligand binding affinity via structural-based drug development practices. It was revealed that π–π interaction between aromatic ring of the ligands and W550 significantly enhanced the binding affinity of the ligand to CSF1R kinase domain. Since several TKIs were developed based on a pyrimidine core due to its electron-rich aromatic heterocycle structure is obliged to many human RTKs^87^, the π–π stack interaction between the aromatic ring of W550 and aromatic structure of the inhibitor should be considered in CSF1R-structure based design drug process.

Aromatic-aromatic interactions greatly contribute to protein stability, protein–ligand interactions, catalysis, among other protein activities in cells, as they play a major role in chemical and biological recognition^88–91^. A typical π–π interaction has an energy between − 0.5 to − 2.0 kcal/mol^92^. The π–π interactions, together with hydrogen bonds, are commonly involved in anchoring drugs in many proteins and importantly contribute to the ligand binding affinity^91–94^. In an auto-inhibit state, the CSF1R residue W550 interacts with the carbonyl group of D796 thus stabilizing the DFG-out conformation of the activation loop, maintaining CSF1R in a catalytically inactive state which opens an allosteric pocket adjacent to the ATP-binding pocket. The allosteric pocket located in the kinase domain of the receptor. This allosteric pocket, together with the inward ATP binding site, is the classical binding pocket of the type II TKIs. One of the important interacted residues commonly found in CSF1R-inhibitor complex is W550 in the JM region of the kinase. Functionally, the residue is crucial for maintaining the autoinhibitory state of CSF1R by preventing the activation loop switch to an active conformation. Many CSF1R inhibitors were also designed based on the pyrimidine-core^87,95–97^ which contain an aromatic ring that favorably form π–π interactions with the aromatic side chain in CSF1R binding pocket, for example, with W550^54,83–87^.

### Kusunokinin and W550 binding possibility

*Trans*-(−)-kusunokinin, isolated naturally from *Piper nigrum*^55^, has structure resemble to those pyrimidine ring-core inhibitors. It inhibits cancer cell growth, proliferation, and metastasis in a variety of cancer cell lines, including breast, colon and lung cancer cell lines^55^. The compound also reduces the size of breast tumours in mouse models without affecting body weight, internal organs, or bone marrow^56^. According to in silico molecular docking studies and in vitro si-RNA studies, *trans*-(−)-kusunokinin targeted CSF1R, inhibiting the receptor and AKT signalling cascade, and thus suppressing cancer cell proliferation and metastasis^53,54^.

A racemic mixture of *trans*-(−)-kusunokinin and *trans*-(+)-kusunokinin was obtained from the synthesis process, however, *trans*-(+)-kusunokinin, in contrast with its *trans*-(−)-isomer, showed no inhibitory effects against cancer cells^98^. Nonetheless, the racemic mixture *trans*-(±)-kusunokinin retained anticancer activities against breast, colon, cholangiocarcinoma^57^ and ovarian cancer cell lines^58^ with low toxicity against normal cells^59^. Therefore, *trans*-(−)-kusunokinin was hypothesised to be the main active component in the racemic mixture, targeting protein in proliferation and metastasis pathway, while *trans*-(+)-kusunokinin may additionally inhibited other related molecules^54^, given that the mechanism of action between *trans*-(−)-kusunokinin, *trans*-(±)-kusunokinin, and CSF1R inhibitor pexidartinib were different^53^.

Previously, the stereoselectivity of CSF1R for *trans*-(−)-kusunokinin over *trans*-(+)-kusunokinin was proposed due to (1) the CSF1R narrow binding pocket requiring planar arrangement of the binding ligand and (2) the π–π interaction between the W550 and the aromatic ring of the ligand being critical to form a stable ligand-CSF1R complex^54^. In both previous study^54^ and this current study, Molecular docking and MD simulations demonstrated that *trans*-(+)-kusunokinin could not fit into the binding pocket of CSF1R and thus could not form an interaction with W550, its binding affinity was much lower than that of *trans*-(−)-kusunokinin.

Taken together, we therefore continue our studies by utilizing in silico methods to further explore the significance of CSF1R W550 to the ligand binding affinity and the impact of this residue on CSF1R conformational change upon ligand binding, given that W550 regulates CSF1R activation structurally by interacting with the carbonyl group of the Aspartic acid position 796th (D796) of the DFG-motif, which stabilises the DFG-out conformation of the activation loop and thus keeps CSF1R inactive^63,74,99^. *Trans*-(−)-kusunokinin, as well as other inhibitors may stabilise the ligand-CSF1R complex and lock CSF1R in an inactive state via binding W550, among other residues. A shorter MD simulation time than in our previous study^54^ was used to ensure that the ligand remained within the binding site throughout the simulations, as we wanted to look at the interactions and conformational changes that occur when binding.

The possible stereoselectivity of CSF1R which prefers *trans*-(−)-kusunokinin to *trans*-(+)-kusunokinin was reported in^54^. In short, it was previously found that *trans*-( ±)-kusunokinin racemic mixture exhibited anticancer activities on breast cancer cell lines via partially bound and inhibited CSF1R^53^. The in silico simulations showed that the *trans*-(–) isomer bound CSF1R with stronger binding energy than the *trans*-(+) isomer and the complex of *trans*-(−)-kusunokinin-CSF1R was also more stable than *trans*-(+)-kusunokinin-CSF1R complex due to *trans*-(−)-kusunokinin forming π–π interaction with CSF1R W550's aromatic ring while *trans*-(+)-kusunokinin did not^54^. The work concluded that (1) W550 is the selective residue determine the kusunokinin enantiomer residence time within the CSF1R binding pocket and (2) the *trans*-(–) isomer was the main active component within the *trans*-(±)-kusunokinin racemic mixture which exhibited anticancer activities via binding and inhibition of CSF1R, while *trans*-(+) isomer possibly targeted additional molecules in breast cancer progression pathways. In this study, we aim to elucidate the impact of interacted residue(s) on the ligand-receptor binding features including binding affinity, target specificity, as well as the ligand-receptor structural-activity relationships.

### Wild type and W550A structural effect due to ligand binding

Nevertheless, the Alanine mutagenesis and the Molecular Dynamics (MD) simulations results demonstrated that W550 might not played important roles in CSF1R conformational change upon ligand binding, not in term of ligand binding features nor crucial for locking CSF1R into an inactive state. Substituting W550 with Alanine which omits of its aromatic side chain did not affect the movement of DFG-motif which indicate active or inactive state of CSF1R. Although minor conformational changes upon mutation were detected, they were not enough to cause ligand unbound from its original binding pocket. All tested compounds still bound CSF1R in inactive state without significantly structural differed from the WT.

There is currently no report on W550 mutation in CSF1R. The residue is conserved among type-III RTKs, except for FLT-3 which has Leucine instead of Tryptophan^82^. W550 is a critical residue for maintaining the autoinhibitory state of CSF1R by interacting with the carbonyl group of D796 thus preventing the activation loop switching to an active conformation. The stabilized DFG-out conformation of the activation loop consequently maintains CSF1R in a catalytically inactive state which opens an allosteric pocket adjacent to the ATP-binding pocket^63,86,100^. Therefore, by removing W550 side chain, CSF1R may be unable to maintain its auto-inactive state hence hyperactivity. However, from the predicted protein stability calculated from FoldX, the mutation W550A could lead to destabilizing effects on the protein, but not enough to cause protein miss-folding or conformation changes in ligand-free state, the CSF1R^W550A^ was still in an auto-inhibit state as the template model CSF1R^WT^, as the alignment results showed in Figs. 4 and 5.

However, MM/GBSA results showed that W550A indeed affected the binding energies of *trans*-(−)-kusunokinin, *trans*-(+)-kusunokinin and imatinib significantly while not affected the binding energy of pexidartinib. The binding energy of *trans*-(−)-kusunokinin was significantly worsen upon mutation, indicated that *trans*-(−)-kusunokinin binding character was abundantly rely on the π–π stack interaction between its benzene ring and W550. In contrast, binding energies of *trans*-(+)-kusunokinin and imatinib were improved upon mutation. Since W550 is a conserved residue in TKR type III^63^, it is not surprising that the disappearance of W550 could affect imatinib binding affinity, despite that imatinib is a pan-inhibitor.

On the other hand, pexidartinib originally bound CSF1R^WT^ with π–π stack interactions both at W550 and F797, arranged its binding pose from the allosteric pocket to ATP-binding site. In addition, the fluorine group at the ligand’s tail also formed halogen bonds with Valine position 548^th^ hence increased binding affinity and secure the ligand in binding pocket without need for W550 π–π stack interaction. With these factors, it is not surprising that mutation W550A showed no significant impact on pexidartinib.

According to MM/GBSA results and interaction analysis in both CSF1R^WT^ and CSF1R^W550A^ with respect to the *trans*-(−)-kusunokinin or drugs, W550, among other factors, is crucial for ligand binding CSF1R. However, in order to improve CSF1R binding affinity, as well as specificity and selectivity, length extension of the ligand with polar groups, mimic to the structure of pexidartinib and imatinib, should be examined. Further study on CSF1R-inhibitor structure–activity relationships (SAR) as well as ADME profile of the modified *trans*-(−)-kusunokinin based CSF1R-specific targeted drugs should be performed according to the proposed guideline noted here.

## Conclusion

W550 plays an important role in regulating CSF1R inhibitory state via the interaction with conserved D796 of the DFG-motif. However, replacing W550 with Alanine had no effect on the binding position of *trans*-(−)-kusunokinin or the CSF1R inhibitors. CSF1R conformational change upon ligand-binding which indicates the inhibitory effect of the binding ligand also unaffected by lack of W550’s aromatic sidechain. Instead, the presence of W550 is important in increasing binding affinity by forming π–π stack interaction with the aromatic structure of the inhibitor. *Trans*-(−)-kusunokinin bound the CSF1R inhibitor site with significantly decreased binding affinity in CSF1R^W550A^, compared to CSF1R^WT^, while the *trans*-(+)-kusunokinin bound differently. Apart from the binding and specificity, the study of off-target effects of the (–)-kusunokinin such as cytotoxicity and drug-likeness gave the acceptable drug-candidate possibility. As a result, to create CSF1R-specific small-molecule inhibitors based on the *trans*-(−)-kusunokinin structure, the benzine ring at one arm should be preserved while the length extension of the ligand with polar groups for increased CSF1R specificity and binding affinity should be considered.

## Methods

### Protein structure preparation

CSF1R kinase 3D X-ray crystal structures in autoinhibited state (PDB ID 2OGV and 8CGC), DFG-in conformation (PBB ID 3LCD), DFG-out conformation (PDB ID 3LCO), and complex with FDA-approved inhibitor pexidartinib (PDB ID 4R7H) were obtained as PDB files from the Protein Data Bank (https://www.rcsb.org). The co-crystallised ligand and solvent molecules were removed using Visual Molecular Dynamics (VMD) software version 1.9.3^101^. All polar hydrogens were added to the CSF1R structures corresponding to the protonation state at pH 7 using AutoDock Auxillary Tool (ADT) version 1.5.6^102^.

### Ligand preparation

The co-crystallised ligands from CSF1R kinase crystal structures were retrieved from the original PDB files to be docked as reference binding sites to compare with the tested ligand. The 3D conformers of *trans*-(−)-kusunokinin and *trans*-(+)-kusunokinin, as well as 14 pan-RTK class III inhibitors and 15 specific/dual CSF1R antagonists (name and CID listed in Table S1) were obtained from the PubChem database (https://pubchem.ncbi.nlm.nih.gov). SDF files were converted to PDB using the Online SMILE Translator and Structure File Generator (https://cactus.nci.nih.gov/translate/index.html). Missing polar hydrogens were added to the ligand structures and saved as PDBQT file format using AutoDock Auxillary Tool (ADT) version 1.5.6 prior to molecular docking.

### Molecular docking

Molecular docking was performed on AutoDock version 4.2.6^102^. All docking parameters were set based on previous studies^54,103^ using python shell command from MGLTools version 1.5.4^104^. The grid box was set on the center of the macromolecule with x–y–z grid point numbers of 126–126–126, which was the maximum grid box size to ensure the grid box covered the whole protein structure. The grid spacing was set at 0.375 Å as a default value (Fig. S5). A genetic algorithm (GA) with 50 runs and a population size of 200 was performed with default parameters. A Lamarckian genetic algorithm (4.2) was applied to predict binding conformations. The docking procedure was run 5 times for each system. Only the binding poses of the docked ligand within the same binding pocket as the reference co-crystallised CSF1R inhibitors were accepted for further investigation as described in previous study^54^. The lowest docking score in kcal/mol was considered the best binding pose. The Biovia Discovery Studio Visualizer version 19.1^105^ and VMD were used to see the ligand–protein interactions and docking poses.

### Alanine mutagenesis

The structure of CSF1R kinase in complex with pexidartinib (PDB ID 4R7H) was used as a CSF1R wild-type (CSF1R^WT^) template to generate mutated CSF1R^W550A^ model with the FoldX^106^ BuildModel command. The mutated models were then optimized and folding-free energy changes upon mutation in kcal/mol were calculated using the Stability command to evaluate the protein stability upon point mutation^64^. All models were superimposed, and conformational changes were examined prior to the docking procedure.

After generated the mutated model, it was optimized using the *Optimize* command in FoldX version 5.0^106^ to repair any missing side chain atoms and eliminate the Vander Waals clashes upon the introduced residue substitution. The folding-free energy changes upon mutation (ΔΔG) in kcal/mol were calculated using the *Stability* command to evaluate the protein stability upon point mutation. The ΔΔG between − 0.75 and − 5 kcal/mol was considered to have stabilizing effects and ΔΔG of >  + 1 kcal/mol was considered to have destabilizing effects on the mutated model^64,107^. The mutated model was superimposed to the wild-type 4R7H template using VMD to examine the conformational changes upon mutation prior to the docking procedure. The mutated model without significant conformational changes which threaten to docking failures was accepted to perform Molecular docking and MD simulations.

### Molecular dynamics (MD) simulations

The docked ligand–protein complexes served as the starting points for subsequent MD runs with the AMBER16 package^108^. The system preparation and minimization steps were carried out at 298 K and 1.013 Bar in an isotonic 0.15 M NaCl solution, as previously studied^54^. The simulation was run for 100 ns in triplicate, which equivalate to a total of 300 ns sampling time per model. We decided to perform independent 100-ns MD simulations in triplicate^109–111^ to reduce the bias from the initial structure and to ensure the ligand stays within the binding pocket, as an increasing instability of *trans*-(+)-kusunokinin-CSF1R complex was observed after 100 ns from the total 300 ns simulation study^54^.

Using the root mean square deviation (RMSD) parameter, the MD trajectory of 2000 snapshots was examined for ligand–protein stability. The last 800 snapshots were used to evaluate conformational changes caused by ligand binding using the root-mean square fluctuation (RMSF) based on the fluctuation of the amino acid α-carbon position. The amino number in this paper was referred to the amino sequence number of human CSF1R (ID 4R7H), concluded in the Table S4. To ensure the simulated system reaches the equilibrium state, the NVT ensemble temperature fluctuating, the RMSD and the radius of gyration (Rg) were computed and the deviation of the 100-ns simulation temperature, the RMSD and the Rg between the triplicated runs were calculated.

The average relative binding free energies (ΔG) in kcal/mol, as well as the total contribution energies of interacted residues, were also calculated from the triplicated last 800 snapshots using molecular mechanics/generalized Born-surface area (MM/GBSA)^79^. The energetics terms were briefly summarised in the previous studies^54,80,81^. All the MD results in triplicate, in the total of 300 ns sampling, from each system in three independent simulations were calculated with LibreOffice Calc version 5.1.6.2 and represented as mean ± SD. Ubuntu Xmgrace version 5.1.25 was used to visualise the graph. All software was operated in Ubuntu version 16.04.

### Distance analysis

To determine the ligand's binding profiles to the protein, distance between the ligand center of mass and the protein center of mass was averaged by the triplicated MD system and plotted along the simulation to investigate the effect of the W550A mutation on ligand binding pocket.

**Figure 8:**
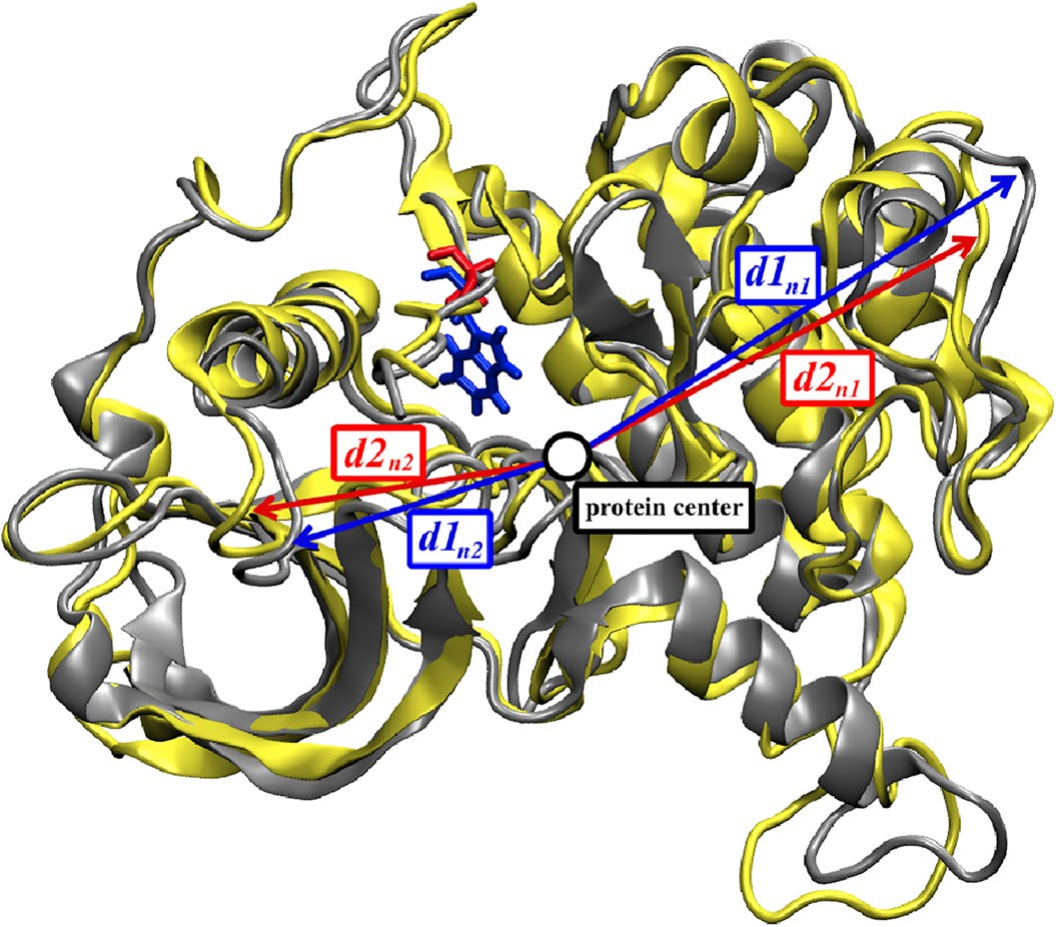
Concept for Distance Analysis. In the wild-type simulation, the distances from the center of structure to amino residues **1** and **2** are denoted by *d1*_*n1*_ and *d1*_*n2*_, whereas in the W550A simulation, the distances are denoted by *d2*_*n1*_ and *d2*_*n2*_. The varying distances indicated the position of the same amino acid in both wild-type and mutant structures. If *d1*_*n1*_ is longer than *d2*_*n1*_, it indicates that amino residue **1** is further from the center, vice versa. In this concept, a significant difference in distance may indicate that the amino residue is in a different position as well as conformational similarity. The protein structures were generated using Visual Molecular Dynamics (VMD) software version 1.9.3.

The distances from the protein center to each amino acid residue were also retrieved from the last 800 snapshots and were averaged by the triplicated MD system. The conformational changes upon ligand binding and the mutation impact on this aspect were measured by comparing the differences in the average distance patterns (Δ*d*) between ligand-free CSF1R^WT^ and ligand-free CSF1R^W550A^. The Δ*d* between the ligand-free CSF1R^WT^ and the ligand-bound CSF1R^WT^ (Δ*d*_1_) or the Δd between the ligand-free CSF1R^W550A^ and the ligand-bound CSF1R^W500A^ (Δ*d*_2_) were also measured to indicate the conformational changes upon ligand-binding. The Δ*d*_1_ and the Δ*d*_2_ were plotted together to visualize the impact of alanine mutagenesis in term of ligand-binding to protein and the differences in the Δ*d*_1_ and the Δ*d*_2_ (ΔΔ*d*) were calculated to determine the effected residue. The absolute ΔΔ*d* greater than 1.5 Å were considered effected, Fig. 8.

To indicate the inhibitory effect of tested ligand, the α-carbon distance between CSF1R Asparagine position 783rd (D783) which is the first Asparagine follows the HRDxxxN motif and Phenylalanine position 797th (F797) in the DFG motif (*d*_1_) was compared to the α-carbon distance between the conserved Glutamic acid position 633rd (E633) on the αC-helix and Phenylalanine position 797th (F797) (*d*_2_). The CSF1R state was considered DFG-out or inactive state when *d*_1_ < *d*_2_ and considered DFG-in or active state when *d*_1_ > *d*_2_. The classical DFG-out conformation cut-off at *d*_1_ < 7.2 Å and *d*_2_ > 9 Å^43^.

### Off-target predictions

The off-target predictions were performed using Protox 3.0 (https://comptox.charite.de/protox3) for cytotoxicity profile, SwissADME (http://www.swissadme.ch/) for drug-likeness parameters, and SwissTargetPrediction (http://www.swisstargetprediction.ch/) for drug-target prediction. The input for all predictions stated earlier was the compound isomeric SMILE from Pubchem database. The pubchem CIDs were summarized in the Table S1.

### Supplementary Information

Supplementary Information.

## Data Availability

All data generated or analyzed during the current study are included in this published article or its supplementary data files. The supporting information as the zip files, include 6 tables, and 5 figures as followed: Table S1. Docked ligand name and PubChem CID. Table S2. Docking scores of all tested ligands to all tested CSF1R states. Table S3. Interacted residues of docked ligands. Table S4. Reference- and MD rearranged-residue number of the CSF1R kinase domain PDB ID 4R7H. Table S5. Different in distance from protein center to each amino acid residue (average from the last 800 snapshots from the tripicate MD systems). Table S6. Distance between ASN783 to PHE797 (d1) and GLU633 to PHE797 (d2). Figure S1. Off-target profiles of imatinib. Figure S2. Off-target profiles of pexidartinib. Figure S3. Off-target profiles of *trans*-(–)-kusunokinin. Figure S4. Off-target profiles of *trans*-(+)-kusunokinin. Figure S5. Molecular docking parameters. The off-target profile figures were generated using Protox 3.0 (comptox.charite.de/protox3) for cytotoxicity profile, SwissADME (www.swissadme.ch) for drug-likeness parameters, and SwissTargetPrediction (www.swisstargetprediction.ch) for drug-target prediction.

## Abbreviations

CSF1R: Colony stimulating factor 1 receptor
FDA: Food and drug administration
MD: Molecular dynamics
RTK: Receptor tyrosine kinase
TK: Tyrosine kinase
TKI: Tyrosine kinase inhibitor
