# Supplementary material for: Role of CSF1R 550th-tryptophan in kusunokinin and CSF1R inhibitor binding and ligand-induced structural effect
Source: Sci Rep. 2024 May 31;14:12531. doi: 10.1038/s41598-024-63505-x (PMC11143223; doi:10.1038/s41598-024-63505-x)
Supplement: Supplementary file 1 — Supplementary Information. [file 41598_2024_63505_MOESM1_ESM.zip › Table-S4-Reference-and-MD-rearranged-residue.pdf]

Table S4. Reference- and MD rearranged-residue number of the CSF1R kinase domain PDB ID 4R7H.

Reference residue number	MD residue number	Amino acid abbreviates	Reference residue number	MD residue number	Amino acid abbreviates
544	1	PRO	594	46	GLY
545	2	LYS	595	47	LYS
546	3	TYR	596	48	VAL
547	4	GLN	597	49	VAL
548	5	VAL	598	50	GLU
549	6	ARG	599	51	ALA
550	7	TRP	600	52	THR
551	8	LYS	601	53	ALA
552	9	ILE	602	54	PHE
553	10	ILE	603	55	GLY
554	11	GLU	604	56	LEU
555	12	SER	605	57	GLY
561	13	TYR	606	58	LYS
562	14	THR	607	59	GLU
563	15	PHE	608	60	ASP
564	16	ILE	609	61	ALA
565	17	ASP	610	62	VAL
566	18	PRO	611	63	LEU
567	19	THR	612	64	LYS
568	20	GLN	613	65	VAL
569	21	LEU	614	66	ALA
570	22	PRO	615	67	VAL
571	23	TYR	616	68	LYS
572	24	ASN	617	69	MET
573	25	GLU	618	70	LEU
574	26	LYS	619	71	LYS
575	27	TRP	620	72	SER
576	28	GLU	621	73	THR
577	29	PHE	622	74	ALA
578	30	PRO	623	75	HIS
579	31	ARG	624	76	ALA
580	32	ASN	625	77	ASP
581	33	ASN	626	78	GLU
582	34	LEU	627	79	LYS
583	35	GLN	628	80	GLU
584	36	PHE	629	81	ALA
585	37	GLY	630	82	LEU
586	38	LYS	631	83	MET
587	39	THR	632	84	SER
588	40	LEU	633	85	GLU
589	41	GLY	634	86	LEU
590	42	ALA	635	87	LYS
591	43	GLY	636	88	ILE
592	44	ALA	637	89	MET
593	45	PHE	638	90	SER

Table S4. *Cont.*

Reference residue number	MD residue number	Amino acid abbreviates	Reference residue number	MD residue number	Amino acid abbreviates
639	91	HIS	684	136	GLY
640	92	LEU	685	137	PRO
641	93	GLY	686	138	SER
642	94	GLN	687	139	LEU
643	95	HIS	747	140	GLY
644	96	GLU	748	141	ARG
645	97	ASN	749	142	PRO
646	98	ILE	750	143	LEU
647	99	VAL	751	144	GLU
648	100	ASN	752	145	LEU
649	101	LEU	753	146	ARG
650	102	LEU	754	147	ASP
651	103	GLY	755	148	LEU
652	104	ALA	756	149	LEU
653	105	CYS	757	150	HIS
654	106	THR	758	151	PHE
655	107	HIS	759	152	SER
656	108	GLY	760	153	SER
657	109	GLY	761	154	GLN
658	110	PRO	762	155	VAL
659	111	VAL	763	156	ALA
660	112	LEU	764	157	GLN
661	113	VAL	765	158	GLY
662	114	ILE	766	159	MET
663	115	THR	767	160	ALA
664	116	GLU	768	161	PHE
665	117	TYR	769	162	LEU
666	118	CYS	770	163	ALA
667	119	THR	771	164	SER
668	120	TYR	772	165	LYS
669	121	GLY	773	166	ASN
670	122	ASP	774	167	CYS
671	123	LEU	775	168	ILE
672	124	LEU	776	169	HIS
673	125	ASN	777	170	ARG
674	126	PHE	778	171	ASP
675	127	LEU	779	172	VAL
676	128	ARG	780	173	ALA
677	129	ARG	781	174	ALA
678	130	LYS	782	175	ARG
679	131	ALA	783	176	ASN
680	132	GLU	784	177	VAL
681	133	ALA	785	178	LEU
682	134	MET	786	179	LEU
683	135	LEU	787	180	THR

Table S4. *Cont.*

Reference residue number	MD residue number	Amino acid abbreviates	Reference residue number	MD residue number	Amino acid abbreviates
788	181	ASN	833	226	THR
789	182	GLY	834	227	VAL
790	183	HIS	835	228	GLN
791	184	VAL	836	229	SER
792	185	ALA	837	230	ASP
793	186	LYS	838	231	VAL
794	187	ILE	839	232	TRP
795	188	GLY	840	233	SER
796	189	ASP	841	234	TYR
797	190	PHE	842	235	GLY
798	191	GLY	843	236	ILE
799	192	LEU	844	237	LEU
800	193	ALA	845	238	LEU
801	194	ARG	846	239	TRP
802	195	ASP	847	240	GLU
803	196	ILE	848	241	ILE
804	197	MET	849	242	PHE
805	198	ASN	850	243	SER
806	199	ASP	851	244	LEU
807	200	SER	852	245	GLY
808	201	ASN	853	246	LEU
809	202	TYR	854	247	ASN
810	203	ILE	855	248	PRO
811	204	VAL	856	249	TYR
812	205	LYS	857	250	PRO
813	206	GLY	858	251	GLY
814	207	ASN	859	252	ILE
815	208	ALA	860	253	LEU
816	209	ARG	861	254	VAL
817	210	LEU	862	255	ASN
818	211	PRO	863	256	SER
819	212	VAL	864	257	LYS
820	213	LYS	865	258	PHE
821	214	TRP	866	259	TYR
822	215	MET	867	260	LYS
823	216	ALA	868	261	LEU
824	217	PRO	869	262	VAL
825	218	GLU	870	263	LYS
826	219	SER	871	264	ASP
827	220	ILE	872	265	GLY
828	221	PHE	873	266	TYR
829	222	ASP	874	267	GLN
830	223	SER	875	268	MET
831	224	VAL	876	269	ALA
832	225	TYR	877	270	GLN

Table S4. *Cont.*

Reference residue number	MD residue number	Amino acid abbreviates	Reference residue number	MD residue number	Amino acid abbreviates
878	271	PRO	897	290	PRO
879	272	ALA	898	291	THR
880	273	PHE	899	292	HIS
881	274	ALA	900	293	ARG
882	275	PRO	901	294	PRO
883	276	LYS	902	295	THR
884	277	ASN	903	296	PHE
885	278	ILE	904	297	GLN
886	279	TYR	905	298	GLN
887	280	SER	906	299	ILE
888	281	ILE	907	300	THR
889	282	MET	908	301	SER
890	283	GLN	909	302	PHE
891	284	ALA	910	303	LEU
892	285	CYS	911	304	GLN
893	286	TRP	912	305	GLU
894	287	ALA	913	306	GLN
895	288	LEU	914	307	ALA
896	289	GLU	915	308	GLN

The kinase domain of CSF1R is located at residue number 582 – 873 (MD number 34 – 266). The juxtamembrane (JM) region is from residue number 544 – 574 (MD number 1 – 26). The activation loop (AL) region is from residue number 796 – 818 (MD number 189 – 211).
