# Supplementary material for: Role of CSF1R 550th-tryptophan in kusunokinin and CSF1R inhibitor binding and ligand-induced structural effect
Source: Sci Rep. 2024 May 31;14:12531. doi: 10.1038/s41598-024-63505-x (PMC11143223; doi:10.1038/s41598-024-63505-x)
Supplement: Supplementary file 1 — Supplementary Information. [file 41598_2024_63505_MOESM1_ESM.zip › Table-S3-Interacted-residues-of-docked-ligands.pdf]

Table S3. Interacted residues of docked ligands

Pan TKIs-CSF1R kinase (4R7H)			
**Compound**	**Docking score**	**H-bond**	**Pi-Pi / Pi-T**
Chiauranib	-12.96	ARG549<br>GLU664	TRP550
Pazopanib	-11.95	TRP550<br>GLU633<br>GLU664<br>ASP796	TRP550<br>PHE797
Quizartinib	-11.54		PHE797
Sorafenib	-11.46	ARG549<br>THR663<br>CYS666<br>ASP796	TRP550<br>PHE797
Imatinib	-11.16	LEU588<br>ASP670	TRP550<br>TYR665<br>PHE797
Linifanib	-10.99	LYS616<br>GLU664	TRP550
Nilotinib	-10.58	ASP796<br>GLU633<br>LYS616	
Sunitinib	-10.11	THR663<br>CYS666<br>ASP796	PHE797
Dasatinib	-9.93	LEU588<br>CYS666<br>THR663<br>GLU664	
Tadutinib	-9.67	CYS666<br>ASP670<br>ARG801	TYR665
OSI-930	-9.48	THR663<br>CYS666	TRP550<br>PHE797
Sulfatinib	-9.15	TYR546<br>GLU633<br>THR663<br>GLU664<br>ASP796	PHE797
Dovitinib	-8.95	CYS666	TYR665
Tinengotinib	-8.34	TYR665<br>CYS666<br>THR667	

Average docking scores within group = -10.4479 kcal/mol. Double line represented cut-off at the average docking scores among groups (-10.0871 kcal/mol). Docked ligands with docking scores above the cut-off were considered higher binding affinity than docked ligands below the cut-off.

Table S3. *Cont.*

Specific CSF1R inhibitors-CSF1R kinase (4R7H)			
**Compound**	**Docking score**	**H-bond**	**Pi-Pi / Pi-T**
BRP1R024	-11.67	ARG549<br>TRP550<br>GLU633<br>GLU664<br>CYS666<br>ASP796	TRP550
Pexidartinib	-11.07	ARG549<br>TRP550<br>GLU633<br>MET637<br>GLU664<br>CYS666	TRP550<br>PHE797
Sotuletinib	-10.55	GLU664<br>ASP796	TRP550
Ki20227	-10.20	GLU633<br>CYS666<br>ASP796	TRP550
PLX5622	-9.96	GLU664<br>CYS666<br>ASP796	
IACS-9439	-9.90	LEU588<br>GLU664<br>CYS666	PHE797
JNJ-28312141	-9.52	CYS666<br>THR667	TYR665<br>PHE797
JTE-952	-9.51	CYS666<br>ARG782<br>ARG801	PHE797
Pimicotinib	-9.45	CYS666	
Edicotinib	-9.38	THR667	PHE797
Vimseltinib	-9.25	GLU633<br>CYS666<br>ASP670	PHE797
GW2580	-8.87	GLU633<br>CYS666<br>ASP796	PHE797
ARRY-382	-8.75	LEU588<br>CYS666<br>ASP670	TYR665
Q27456873	-8.60	LEU588<br>ASP670<br>ARG801	
AZD7507	-7.97	GLU664<br>CYS666<br>TYR668	TYR665<br>PHE797

Average docking scores within group = -9.6433 kcal/mol. Double line represented cut-off at the average docking scores among groups (-10.0871 kcal/mol). Docked ligands with docking scores above the cut-off were considered higher binding affinity than docked ligands below the cut-off.

Table S3. *Cont.*

***Trans*-(±)-kusunoinin-CSF1R kinase (4R7H)**			
**Compound**	**Docking score**	**H-bond**	**Pi-Pi / Pi-T**
Trans-(–)-kusunokinin	-11.47	ARG549<br>TRP550	TRP550
Trans-(+)-kusunokinin	-10.31	CYS666<br>ASP796	PHE797

Double line represented cut-off at the average docking scores among groups (-10.0871 kcal/mol). Docked ligands with docking scores above the cut-off were considered higher binding affinity than docked ligands below the cut-off.

Frequency

TYR546 = 1 (Sulfetinib)
ARG549 = 5 (All ligands were above the cut-off)
TRP550 = 11 (Ten ligands were above the cut-off, Only OSI-930 was below the cut-off:)
LEU588 = 5
LYS616 = 2
GLU633 = 8
MET637 = 1 (Pexidartinib)
THR663 = 4 (All ligands were pan-TKIs)
GLU664 = 11
TYR665 = 7 (Six formed either π-π or π-T shape, only imatinib was above the cut-off)
CYS666 = 20
TYR668 = 1 (AZD7507)
ASP670 = 5
ARG782 = 1 (JTE-952)
ASP796 = 11
PHE797 =16 (Seven ligands were above the cut-off, nine ligands were below the cut-off)
ARG801 = 3
