# Supplementary material for: Role of CSF1R 550th-tryptophan in kusunokinin and CSF1R inhibitor binding and ligand-induced structural effect
Source: Sci Rep. 2024 May 31;14:12531. doi: 10.1038/s41598-024-63505-x (PMC11143223; doi:10.1038/s41598-024-63505-x)
Supplement: Supplementary file 1 — Supplementary Information. [file 41598_2024_63505_MOESM1_ESM.zip › Table-S2-Docking-scores-of-ligands-to-CSF1R.pdf]

Table S2. Docking scores of all tested ligands to all tested CSF1R states.

Docked ligand	8CGC	2OGV	4R7H	3LCD	3LCO	W550A
Test (2 compounds)						
Trans-(-)-kusunokinin	-8.95	-8.97	-11.47	-8.54	-9.56	-10.08
Trans-(+)-kusunokinin	-9.19	-8.65	-9.87	-8	-9.71	-9.73
Reference (4 compounds)						
Pexidartinib	-7.99	-8.65	-11.07	-7.91	-9.96	-10.44
BDY	-8.44	-9.39	-10.36	-9.64	-11.08	-10.83
LC0	-7.89	-7.93	-10.27	-7.72	-9.64	-9.89
UIK	-8.51	-9.32	-9.69	-8.63	-9.23	-10.58
Pan-TKIs (14 compounds)						
Chiauranib	-11.02	-9.67	-12.96	-9.85	-11.95	-12.71
Dasatinib	-8.09	-7.78	-9.93	-8.86	-10.06	-10.1
Dovitinib	-6.61	-8.27	-8.95	-7.18	-9.27	-8.82
Imatinib	-7.35	-9.46	-11.16	-9.11	-11.74	-11.12
Linifanib	-8.12	-9.13	-10.99	-8.18	-10.2	-11.21
Nilotinib	-8.48	-8.76	-10.58	-9.13	-11.37	-12.04
Osi930	-8.12	-7.95	-9.48	-8.27	-9.51	-10.04
Pazopanib	-8.19	-8.36	-11.95	-9.24	-9.91	-10.34
Quizartinib	-8.98	-9.71	-11.54	-9.6	-12.87	-12.86
Sorafenib	-8.11	-9.96	-11.46	-7.72	-11.26	-10.9
Sulfatinib	-6.62	-7.97	-9.15	-8.06	-9.95	-9.95
Sunitinib	-6.53	-8.36	-10.11	-8.27	-10.5	-10.67
Tandutinib	-7.25	-7.41	-9.67	-8.79	-11.61	-9.54
Tinengotinib	-8.78	-7.18	-8.34	-8.17	-9.16	-9.13
Specific (15 compounds)						
ARRY-382	-7.77	-8.28	-8.75	-9.99	-10.01	-8.3
AZD7505	-7.75	-7.2	-7.97	-8.58	-9.42	-8.15
BRP1R024	-7.4	-7.33	-11.67	-8.29	-10.63	-10.62
Edicotinib	-10.16	-8.91	-9.38	-10.46	-10.59	-9.22
GW2580	-6.91	-8.16	-8.87	-7.47	-9.11	-9.18
IACS-9439	-7.6	-8.59	-9.9	-9.57	-10.63	-9.88
JNJ28312141	-8.9	-8.56	-9.52	-9.42	-10.81	-9.38
JTE-952	-7.46	-6.25	-9.51	-8.08	-9.47	-9.69
KI20227	-8.63	-7.79	-10.2	-8.27	-11.01	-9.75
Pexidartinib	-7.99	-8.65	-11.07	-7.91	-9.96	-10.44
Pimicotinib	-8.28	-8.85	-9.45	-8.57	-10.63	-10.98
PLX5622	-7.93	-8.25	-9.96	-7.89	-9.69	-9.64
Q27456873	-8.76	-8.52	-8.6	-9.04	-11.16	-9.41
Sotuletinib	-8.7	-8.88	-10.55	-8.54	-10.78	-10.68
Vimseltinib	-7.46	-7.87	-9.25	-7.97	-9.51	-9.86

Docking scores represented binding energies calculated from the best binding pose (kcal/mol).
