# Supplementary material for: Role of CSF1R 550th-tryptophan in kusunokinin and CSF1R inhibitor binding and ligand-induced structural effect
Source: Sci Rep. 2024 May 31;14:12531. doi: 10.1038/s41598-024-63505-x (PMC11143223; doi:10.1038/s41598-024-63505-x)
Supplement: Supplementary file 1 — Supplementary Information. [file 41598_2024_63505_MOESM1_ESM.zip › Table-S1-Docked-ligand-name-and-CID.pdf]

Table S1. Docked ligand name and PubChem CID.

No.	Pan TKIs				Selective CSF1R/Dual CSF1R inhibitors			
	**Name**	**CID**	**Phase**	**Reported binding**	**Name**	**CID**	**Phase**	**Reported binding**
1	Sorafenib	216239	3	VEGFR-2, VEGFR-3, PDGFR-β, Flt-3 and c-KIT	Edicotinib (JNJ-527)	25230468	2	Dual CSF1R/KIT
2	Sulfatinib (Surufatunub)	52920501	2	VEGFR1, VEGFR2, VEGFR3, FGFR1 and CSF1R	GW2580	11617559	pre-clinical	
3	Linifanib (ABT-869)	11485656	3	ATP-competitive VEGFR/PDGFR inhibitor for KDR, CSF-1R, Flt-1/3 and PDGFRβ	PLX5622	52936034	1b	
4	Pazopanib	10113978	3	VEGFR1, VEGFR2, VEGFR3, PDGFR, FGFR, c-Kit and c-Fms/CSF1R	BPR1R024	155195529	pre-clinical	
5	Quizartinib (AC220)	24889392	3	Flt3(ITD/WT) 10-fold more selective for Flt3 than KIT, PDGFRα, PDGFRβ, RET, and CSF-1R	ARRY-382	67259771	1b/2	
6	OSI-930	9868037	1	Kit (c-Kit), KDR and CSF-1R	Sotuletinib (BLZ945)	46184986	2	
7	Tinengotinib	137279257	2	Aurora A/B, FGFR1/2/3, VEGFR1/2/3, JAK1/2, and CSF1R	IACS-9439	154025206	pre-clinical	
8	Dovitinib (TKI258, CHIR258)	135398510	4	class III (FLT3/c-Kit)	Pimicotinib (ABSK021)	139549388	3	

Table S1. *Cont.*

9	Chiauranib (CS2164)	49779393	1b/2	VEGFR1, VEGFR2, VEGFR3, PDGFRα and c-Kit, mitosis-related kinase Aurora B, CSF-1R	AZD7507	25001557	pre-clinical	
10	Nilotinib	644241	4	Bcr-Abl tyrosine kinase	Pexidartinib (PLX3397)	25151352	3	Dual CSF1R/KIT
11	Dasatinib	3062316	4	Abl, Src and c-Kit	Vimseltinib (DCC-3014)	86267612	3	Dual CSF1R/KIT
12	Imatinib	5291	4	v-Abl, c-Kit and PDGFR	Ki20227	9869779	pre-clinical	
13	Sunitinib	5329102	4	VEGFR2 (Flk-1) and PDGFRβ	JNJ-28312141	11676971	Obtained from 3KRJ	https://pubs.acs.org/doi/10.1021/jm200900q
14	Tandutinib	3038522	2	FLT3 antagonist, also inhibits PDGFR and c-Kit	JTE-952	49806372	Obtained from 6IG8	https://www.sciencedirect.com/science/article/abs/pii/S0960894X18308527?via%3Dihub
15					Q27456873; 8C5	24938241	Obtained from 3DPK	https://pubs.acs.org/doi/10.1021/jm801406h

CSF1R state	PDB ID	Native ligand	CID
Auto inhibit	2OGV/8CGC	Compound 23; UIK native to 8CGC	168069186
DFG-out	3LCO	Q27462382; LC0 native to 3LCO	46870016
DFG-in	3LCD	Q27458350; BDY native to 3LCD	44631856
Reference for $CSF1R^{WT}$ and $CSF1R^{W550A}$	4R7H	Pexidartinib; P31 native to 4R7H	25151352
