# Supplementary material for: Role of CSF1R 550th-tryptophan in kusunokinin and CSF1R inhibitor binding and ligand-induced structural effect
Source: Sci Rep. 2024 May 31;14:12531. doi: 10.1038/s41598-024-63505-x (PMC11143223; doi:10.1038/s41598-024-63505-x)
Supplement: Supplementary file 1 — Supplementary Information. [file 41598_2024_63505_MOESM1_ESM.zip › Figure-S4-Off-target-profiles-trans-(+)-kusunokinin.pdf]

Figure S4. Off-target profiles of *trans*-(+)-kusunokinin. All off-target predictions were performed using Protox 3.0 for cytotoxicity(https://tox.charite.de/protox3/), SwissADME for drug-likeness(http://www.swissadme.ch/), and SwissTargetPrediction for drug-target prediction (http://www.swisstargetprediction.ch/).

Toxicity Model Report

Copy Excel CSV PDF

Classification	Target	Shorthand	Prediction	Probability
Organ toxicity	Hepatotoxicity	dili	Inactive	0.82
Organ toxicity	Neurotoxicity	neuro	Inactive	0.76
Organ toxicity	Nephrotoxicity	nephro	Active	0.56
Organ toxicity	Respiratory toxicity	respi	Inactive	0.52
Organ toxicity	Cardiotoxicity	cardio	Inactive	0.50
Toxicity end points	Carcinogenicity	carcino	Active	0.52
Toxicity end points	Immunotoxicity	immuno	Active	0.99
Toxicity end points	Mutagenicity	mutagen	Inactive	0.54
Toxicity end points	Cytotoxicity	cyto	Inactive	0.97
Toxicity end points	BBB-barrier	bbb	Active	0.79
Toxicity end points	Ecotoxicity	eco	Active	0.51
Toxicity end points	Clinical toxicity	clinical	Inactive	0.63
Toxicity end points	Nutritional toxicity	nutri	Inactive	0.58

Oral toxicity prediction results for input compound

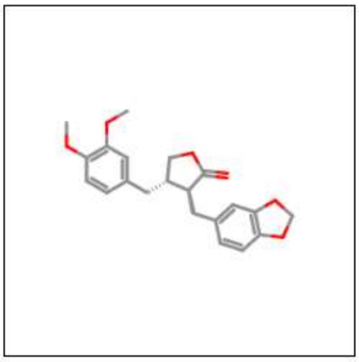

Predicted LD50: 1500mg/kg

Predicted Toxicity Class: 4

1	2	3	4	5	6

Average similarity: 75.75%

Prediction accuracy: 69.26%

20% 40% 60% 80%

Print Toxicity Report

Name	
Molweight	370.4
Number of hydrogen bond acceptors	6
Number of hydrogen bond donors	0
Number of atoms	27
Number of bonds	30
Number of rotable bonds	6
Molecular refractivity	98.15
Topological Polar Surface Area	63.22
octanol/water partition coefficient(logP)	3.01

Comparison of input compound with dataset compounds

Value of input compound
Mean value of dataset

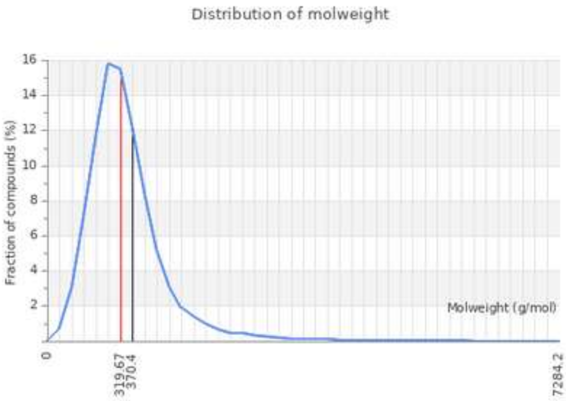

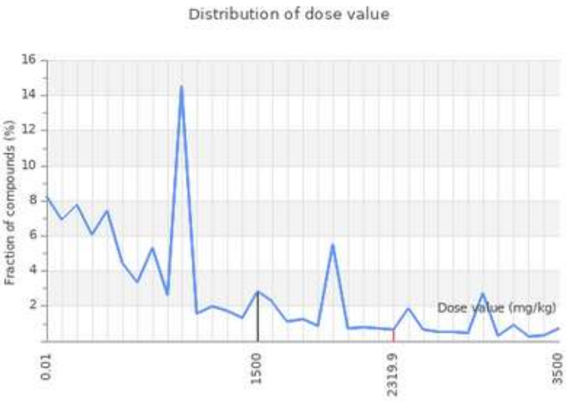

Toxicity targets

Possible binding to toxicity targets is shown below. For more information on the targets, please click on the individual abbreviations.

no binding ... probable binding

AA2AR	ADRB2	ANDR	AOFA	CRFR1	DRD3	ESR1	ESR2	GCR	HRH1	NR1I2	OPRK	OPRM	PDE4D	PGH1	PRGR

Details about possible toxicity targets:

	Toxicity Target	Avg Pharmacophore Fit	Avg Similarity Known Ligands
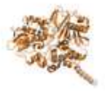	Amine Oxidase A	25.23%	86.14%
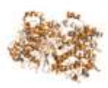	Prostaglandin G/H Synthase 1	65.77%	0%

Last updated: March 2024

Disclaimer: Compound structures submitted will not be released under any circumstances. This work is licensed under a Creative Commons License.

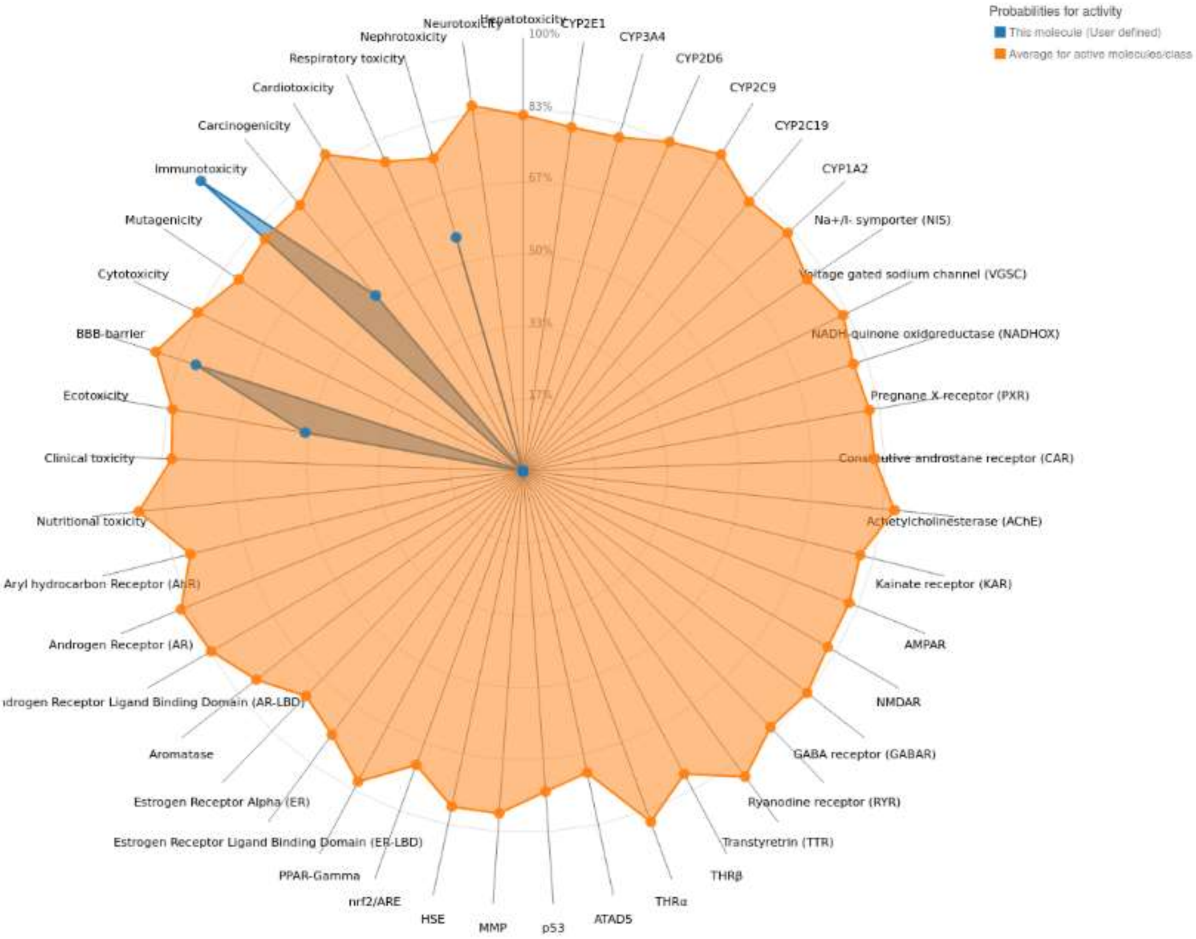

Probabilities for activity
This molecule (User defined)
Average for active molecules/class
100%
83%
67%
50%
33%
17%
Hepatotoxicity
CYP2E1
CYP3A4
CYP2D6
CYP2C9
CYP2C19
CYP1A2
Na+/I- symporter (NIS)
ltage gated sodium channel (VGSC)
NADH-quinone oxidoreductase (NADHOX)
Pregnane X receptor (PXR)
utive androstane receptor (CAR)
Acetylcholinesterase (AChE)
Kainate receptor (KAR)
AMPAR
NMDAR
GABA receptor (GABAR)
Ryanodine receptor (RYR)
Transtyretrin (TTR)
THRβ
THRα
ATAD5
p53
MMP
HSE
nrf2/ARE
PPAR-Gamma
Estrogen Receptor Ligand Binding Domain (ER-LBD)
Estrogen Receptor Alpha (ER)
Aromatase
drogen Receptor Ligand Binding Domain (AR-LBD)
Androgen Receptor (AR)
Aryl hydrocarbon Receptor (AhR)
Nutritional toxicity
Clinical toxicity
Ecotoxicity
BBB-barrier
Cytotoxicity
Mutagenicity
Immunotoxicity
Carcinogenicity
Cardiotoxicity
Respiratory toxicity
Nephrotoxicity
Neurotoxicity

Molecule 1

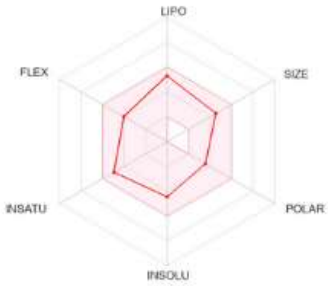

SMILES COc1cc(ccc1OC)C[C@H]1COC(=O)[C@@H]1Cc1ccc2c(c1)OCO2

Physicochemical Properties	
Formula	C21H22O6
Molecular weight	370.40 g/mol
Num. heavy atoms	27
Num. arom. heavy atoms	12
Fraction Csp3	0.38
Num. rotatable bonds	6
Num. H-bond acceptors	6
Num. H-bond donors	0
Molar Refractivity	98.15
TPSA	63.22 Å²

Lipophilicity	
Log $P_{o/w}$ (iLOGP)	3.28
Log $P_{o/w}$ (XLOGP3)	3.78
Log $P_{o/w}$ (WLOGP)	3.01
Log $P_{o/w}$ (MLOGP)	2.53
Log $P_{o/w}$ (SILICOS-IT)	4.28
Consensus Log $P_{o/w}$	3.37

Water Solubility	
Log *S* (ESOL)	-4.45
Solubility	1.31e-02 mg/ml ; 3.54e-05 mol/l
Class	Moderately soluble
Log *S* (Ali)	-4.80
Solubility	5.85e-03 mg/ml ; 1.58e-05 mol/l
Class	Moderately soluble
Log *S* (SILICOS-IT)	-5.96
Solubility	4.11e-04 mg/ml ; 1.11e-06 mol/l
Class	Moderately soluble

Pharmacokinetics	
GI absorption	High
BBB permeant	Yes
P-gp substrate	No
CYP1A2 inhibitor	No
CYP2C19 inhibitor	Yes
CYP2C9 inhibitor	Yes
CYP2D6 inhibitor	Yes
CYP3A4 inhibitor	Yes
Log K_p (skin permeation)	-5.88 cm/s

Druglikeness	
Lipinski	Yes; 0 violation
Ghose	Yes
Veber	Yes
Egan	Yes
Muegge	Yes
Bioavailability Score	0.55

Medicinal Chemistry	
PAINS	0 alert
Brenk	0 alert
Leadlikeness	No; 2 violations: MW>350, XLOGP3>3.5
Synthetic accessibility	3.72

SIB Swiss Institute of Bioinformatics

SwissTargetPrediction

Home FAQ Help Download Contact Disclaimer

Query Molecule

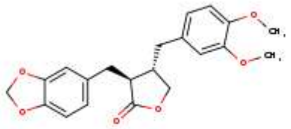

Target Classes

Top 15
Top 25
Top 50
All

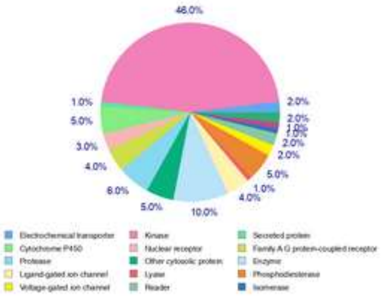

Export results:

Show 15 entries Search:

Target	Common name	Uniprot ID	ChEMBL ID	Target Class	Probability*	Known actives (3D/2D)
Dopamine transporter	SLC6A3	Q01959	CHEMBL238	Electrochemical transporter		50 / 91
Norepinephrine transporter	SLC6A2	P23975	CHEMBL222	Electrochemical transporter		19 / 51
Dual specificity mitogen-activated protein kinase kinase 1	MAP2K1	Q02750	CHEMBL3587	Kinase		103 / 1
Testis-specific androgen-binding protein	SHBG	P04278	CHEMBL3305	Secreted protein		6 / 8
Cytochrome P450 19A1	CYP19A1	P11511	CHEMBL1978	Cytochrome P450		602 / 20
Glucocorticoid receptor	NR3C1	P04150	CHEMBL2034	Nuclear receptor		324 / 10
c-Jun N-terminal kinase 2	MAPK9	P45984	CHEMBL4179	Kinase		177 / 2
Endothelin receptor ET-A	EDNRA	P25101	CHEMBL252	Family A G protein-coupled receptor		17 / 176
Cytochrome P450 11B1	CYP11B1	P15538	CHEMBL1908	Cytochrome P450		295 / 0
Stem cell growth factor receptor	KIT	P10721	CHEMBL1936	Kinase		219 / 0
Cytochrome P450 11B2	CYP11B2	P19099	CHEMBL2722	Cytochrome P450		323 / 0
Cyclin-dependent kinase 5/CDK5 activator 1	CDK5R1 CDK5	Q15078 Q00535	CHEMBL1907600	Kinase		329 / 0
Dual specificty protein kinase CLK1 *(by homology)*	CLK1	P49759	CHEMBL4224	Kinase		140 / 0
Dual specificty protein kinase CLK3 *(by homology)*	CLK3	P49761	CHEMBL4226	Kinase		42 / 0
Cathepsin L	CTSL	P07711	CHEMBL3837	Protease		250 / 0

Showing 1 to 15 of 100 entries

Previous 1 2 3 4 5 6 7 Next

*Probability for the query molecule - assumed as bioactive - to have this protein as target.
