# Supplementary material for: Role of CSF1R 550th-tryptophan in kusunokinin and CSF1R inhibitor binding and ligand-induced structural effect
Source: Sci Rep. 2024 May 31;14:12531. doi: 10.1038/s41598-024-63505-x (PMC11143223; doi:10.1038/s41598-024-63505-x)
Supplement: Supplementary file 1 — Supplementary Information. [file 41598_2024_63505_MOESM1_ESM.zip › Figure-S3-Off-target-profiles-trans-(-)-kusunokinin.pdf]

Figure S3. **Off-target profiles of *trans*-(-)-kusunokinin**. All off-target predictions were performed using Protox 3.0 for cytotoxicity(https://tox.charite.de/protox3/), SwissADME for drug-likeness(http://www.swissadme.ch/), and SwissTargetPrediction for drug-target prediction (http://www.swisstargetprediction.ch/).

Toxicity Model Report

Copy Excel CSV PDF

Classification	Target	Shorthand	Prediction	Probability
Organ toxicity	Hepatotoxicity	dili	Inactive	0.82
Organ toxicity	Neurotoxicity	neuro	Inactive	0.76
Organ toxicity	Nephrotoxicity	nephro	Active	0.56
Organ toxicity	Respiratory toxicity	respi	Inactive	0.52
Organ toxicity	Cardiotoxicity	cardio	Inactive	0.50
Toxicity end points	Carcinogenicity	carcino	Active	0.52
Toxicity end points	Immunotoxicity	immuno	Active	0.99
Toxicity end points	Mutagenicity	mutagen	Inactive	0.54
Toxicity end points	Cytotoxicity	cyto	Inactive	0.97
Toxicity end points	BBB-barrier	bbb	Active	0.79
Toxicity end points	Ecotoxicity	eco	Active	0.51
Toxicity end points	Clinical toxicity	clinical	Inactive	0.63
Toxicity end points	Nutritional toxicity	nutri	Inactive	0.58

Oral toxicity prediction results for input compound

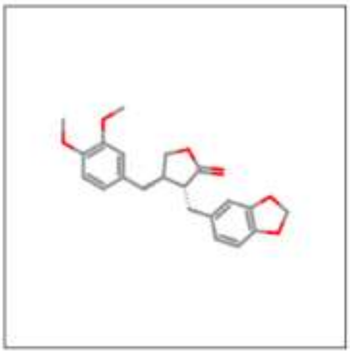

Predicted LD50: 1500mg/kg

Predicted Toxicity Class: 4

1	2	3	4	5	6

Average similarity: 75.75%

Prediction accuracy: 69.26%

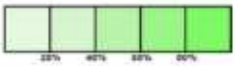

Print Toxicity Report

Name	
Molweight	370.4
Number of hydrogen bond acceptors	6
Number of hydrogen bond donors	0
Number of atoms	27
Number of bonds	30
Number of rotable bonds	6
Molecular refractivity	98.15
Topological Polar Surface Area	63.22
octanol/water partition coefficient(logP)	3.01

Comparison of input compound with dataset compounds

Value of input compound
Mean value of dataset

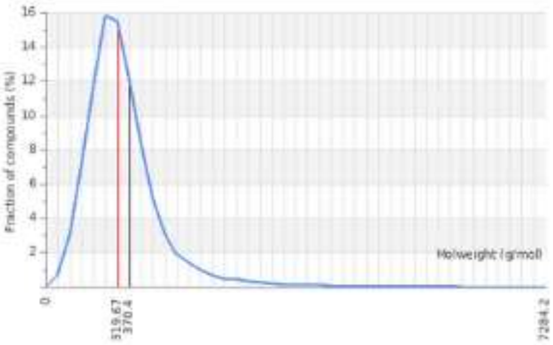

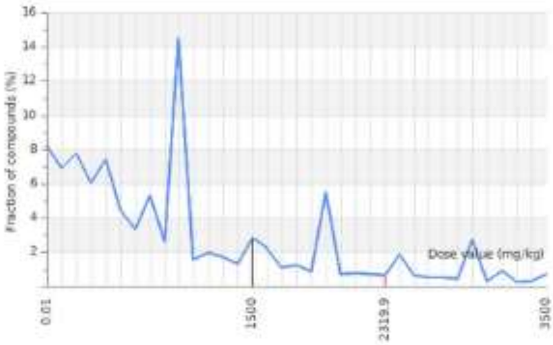

Toxicity targets

Possible binding to toxicity targets is shown below. For more information on the targets, please click on the individual abbreviations.

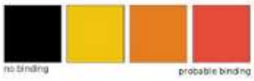

AA2AR	ADRB2	ANDR	AOFA	CRFR1	DRD3	ESR1	ESR2	GCR	HRH1	NR1I2	OPRK	OPRM	PDE4D	PGH1	PRGR

Details about possible toxicity targets:

	Toxicity Target	Avg Pharmacophore Fit	Avg Similarity Known Ligands
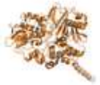	Amine Oxidase A	25.23%	86.14%
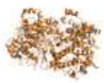	Prostaglandin G/H Synthase 1	65.77%	0%

Last updated: March 2024

Disclaimer: Compound structures submitted will not be released under any circumstances. This work is licensed under a Creative Commons License.

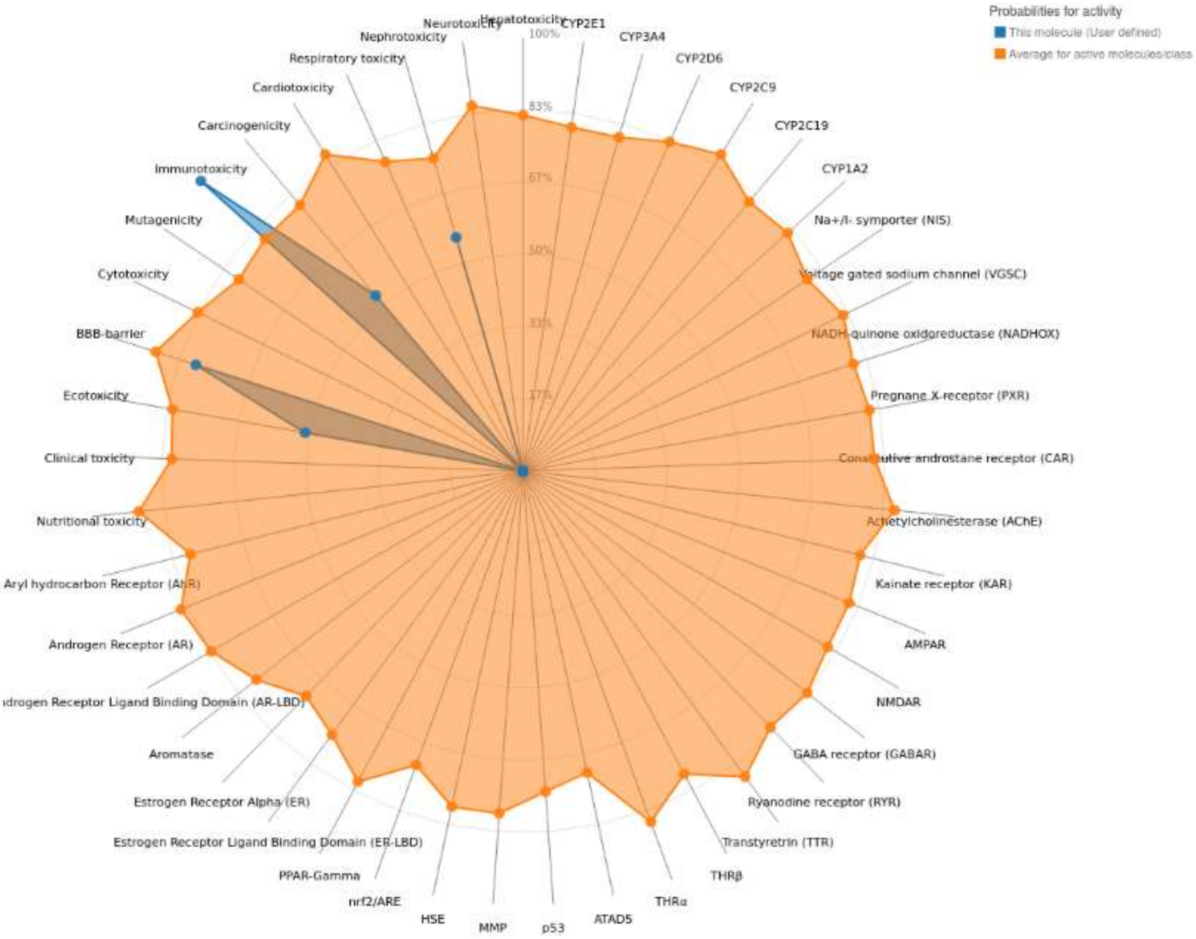

Probabilities for activity
This molecule (User defined)
Average for active molecules/class
Hepatotoxicity
Neurotoxicity
Nephrotoxicity
Respiratory toxicity
Cardiotoxicity
Carcinogenicity
Immunotoxicity
Mutagenicity
Cytotoxicity
BBB-barrier
Ecotoxicity
Clinical toxicity
Nutritional toxicity
Aryl hydrocarbon Receptor (AhR)
Androgen Receptor (AR)
ndrogen Receptor Ligand Binding Domain (AR-LBD)
Aromatase
Estrogen Receptor Alpha (ER)
Estrogen Receptor Ligand Binding Domain (ER-LBD)
PPAR-Gamma
nrf2/ARE
HSE
MMP
p53
ATAD5
THRα
THRβ
Transtyretrin (TTR)
Ryanodine receptor (RYR)
GABA receptor (GABAR)
NMDAR
AMPAR
Kainate receptor (KAR)
Achetylcholinesterase (AChE)
Constitutive androstane receptor (CAR)
Pregnane X receptor (PXR)
NADH-quinone oxidoreductase (NADHOX)
Voltage gated sodium channel (VGSC)
Na+/I- symporter (NIS)
CYP1A2
CYP2C19
CYP2C9
CYP2D6
CYP3A4
CYP2E1
100%
83%
67%
50%
33%
17%

Molecule 1

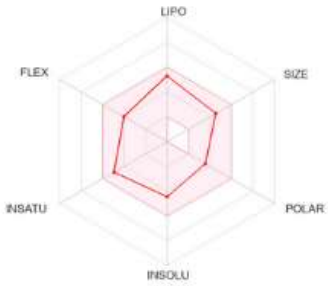

SMILES COc1cc(ccc1OC)C[C@H]1COC(=O)[C@@H]1Cc1ccc2c(c1)OCO2

Physicochemical Properties	
Formula	C21H22O6
Molecular weight	370.40 g/mol
Num. heavy atoms	27
Num. arom. heavy atoms	12
Fraction Csp3	0.38
Num. rotatable bonds	6
Num. H-bond acceptors	6
Num. H-bond donors	0
Molar Refractivity	98.15
TPSA	63.22 Å²

Lipophilicity	
Log $P_{o/w}$ (iLOGP)	3.28
Log $P_{o/w}$ (XLOGP3)	3.78
Log $P_{o/w}$ (WLOGP)	3.01
Log $P_{o/w}$ (MLOGP)	2.53
Log $P_{o/w}$ (SILICOS-IT)	4.28
Consensus Log $P_{o/w}$	3.37

Water Solubility	
Log *S* (ESOL)	-4.45
Solubility	1.31e-02 mg/ml ; 3.54e-05 mol/l
Class	Moderately soluble
Log *S* (Ali)	-4.80
Solubility	5.85e-03 mg/ml ; 1.58e-05 mol/l
Class	Moderately soluble
Log *S* (SILICOS-IT)	-5.96
Solubility	4.11e-04 mg/ml ; 1.11e-06 mol/l
Class	Moderately soluble

Pharmacokinetics	
GI absorption	High
BBB permeant	Yes
P-gp substrate	No
CYP1A2 inhibitor	No
CYP2C19 inhibitor	Yes
CYP2C9 inhibitor	Yes
CYP2D6 inhibitor	Yes
CYP3A4 inhibitor	Yes
Log K_p (skin permeation)	-5.88 cm/s

Druglikeness	
Lipinski	Yes; 0 violation
Ghose	Yes
Veber	Yes
Egan	Yes
Muegge	Yes
Bioavailability Score	0.55

Medicinal Chemistry	
PAINS	0 alert
Brenk	0 alert
Leadlikeness	No; 2 violations: MW>350, XLOGP3>3.5
Synthetic accessibility	3.72

Swiss Institute of Bioinformatics

SwissTargetPrediction

Home FAQ Help Download Contact Disclaimer

Query Molecule

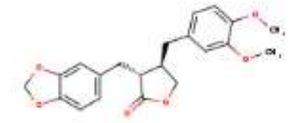

Target Classes

Top 15
Top 25
Top 50
All

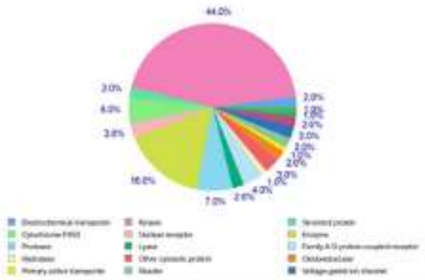

Export results:

Show 15 entries Search:

Target	Common name	Uniprot ID	ChEMBL ID	Target Class	Probability*	Known actives (3D/2D)
Norepinephrine transporter	SLC6A2	P23975	CHEMBL222	Electrochemical transporter		20 / 51
Dopamine transporter	SLC6A3	Q01959	CHEMBL238	Electrochemical transporter		48 / 91
Dual specificity mitogen-activated protein kinase kinase 1	MAP2K1	Q02750	CHEMBL3587	Kinase		96 / 1
Testis-specific androgen-binding protein	SHBG	P04278	CHEMBL3305	Secreted protein		7 / 8
Cytochrome P450 19A1	CYP19A1	P11511	CHEMBL1978	Cytochrome P450		557 / 20
Glucocorticoid receptor	NR3C1	P04150	CHEMBL2034	Nuclear receptor		297 / 10
c-Jun N-terminal kinase 2	MAPK9	P45984	CHEMBL4179	Kinase		163 / 2
Vascular endothelial growth factor receptor 1	FLT1	P17948	CHEMBL1868	Kinase		177 / 0
PI3-kinase p110-alpha/p85-alpha	PIK3CA PIK3R1	P42336 P27986	CHEMBL2111367	Enzyme		17 / 0
Caspase-3	CASP3	P42574	CHEMBL2334	Protease		81 / 0
Caspase-7	CASP7	P55210	CHEMBL3468	Protease		45 / 0
Stem cell growth factor receptor	KIT	P10721	CHEMBL1936	Kinase		224 / 0
Carbonic anhydrase IX	CA9	Q16790	CHEMBL3594	Lyase		793 / 0
Endothelin receptor ET-A	EDNRA	P25101	CHEMBL252	Family A G protein-coupled receptor		18 / 176
Mineralocorticoid receptor	NR3C2	P08235	CHEMBL1994	Nuclear receptor		162 / 0

Showing 1 to 15 of 100 entries

Previous 1 2 3 4 5 6 7 Next
