# Supplementary material for: Role of CSF1R 550th-tryptophan in kusunokinin and CSF1R inhibitor binding and ligand-induced structural effect
Source: Sci Rep. 2024 May 31;14:12531. doi: 10.1038/s41598-024-63505-x (PMC11143223; doi:10.1038/s41598-024-63505-x)
Supplement: Supplementary file 1 — Supplementary Information. [file 41598_2024_63505_MOESM1_ESM.zip › Figure-S2-Off-target-profiles-pexidartinib.pdf]

Figure S2. **Off-target profiles of pexidartinib**. All off-target predictions were performed using Protox 3.0 for cytotoxicity(https://tox.charite.de/protox3/), SwissADME for drug-likeness(http://www.swissadme.ch/), and SwissTargetPrediction for drug-target prediction (http://www.swisstargetprediction.ch/).

Toxicity Model Report

Copy Excel CSV PDF

Classification	Target	Shorthand	Prediction	Probability
Organ toxicity	Hepatotoxicity	dili	Inactive	0.64
Organ toxicity	Neurotoxicity	neuro	Active	0.78
Organ toxicity	Nephrotoxicity	nephro	Inactive	0.79
Organ toxicity	Respiratory toxicity	respi	Active	0.76
Organ toxicity	Cardiotoxicity	cardio	Inactive	0.87
Toxicity end points	Carcinogenicity	carcino	Inactive	0.53
Toxicity end points	Immunotoxicity	immuno	Active	0.68
Toxicity end points	Mutagenicity	mutagen	Inactive	0.58
Toxicity end points	Cytotoxicity	cyto	Active	0.59
Toxicity end points	BBB-barrier	bbb	Active	0.79
Toxicity end points	Ecotoxicity	eco	Active	0.55
Toxicity end points	Clinical toxicity	clinical	Active	0.63
Toxicity end points	Nutritional toxicity	nutri	Active	0.63

Oral toxicity prediction results for input compound

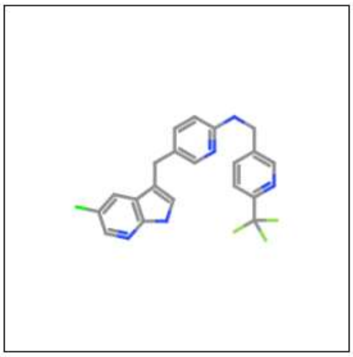

Predicted LD50: 840mg/kg

Predicted Toxicity Class: 4

1	2	3	4	5	6

Average similarity: 48.99%

Prediction accuracy: 54.26%

20% 40% 60% 80%

Name	
Molweight	417.82
Number of hydrogen bond acceptors	4
Number of hydrogen bond donors	2
Number of atoms	29
Number of bonds	32
Number of rotable bonds	6
Molecular refractivity	104.94
Topological Polar Surface Area	66.49
octanol/water partition coefficient(logP)	5.3

Print Toxicity Report

Comparison of input compound with dataset compounds

Value of input compound

Mean value of dataset

Distribution of molweight

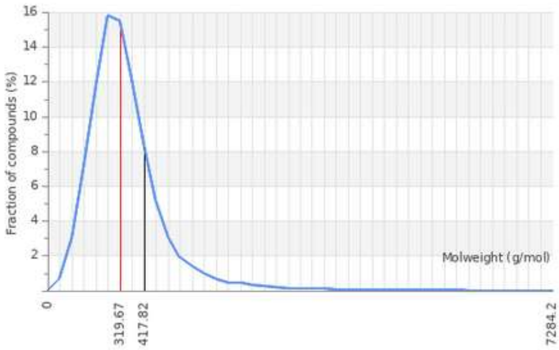

Distribution of dose value

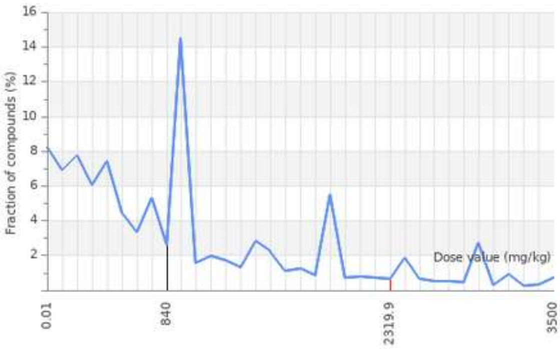

Toxicity targets

Possible binding to toxicity targets is shown below. For more information on the targets, please click on the individual abbreviations.

no binding probable binding

AA2AR	ADRB2	ANDR	AOFA	CRFR1	DRD3	ESR1	ESR2	GCR	HRH1	NR1I2	OPRK	OPRM	PDE4D	PGH1	PRGR

Last updated: March 2024

Disclaimer: Compound structures submitted will not be released under any circumstances. This work is licensed under a Creative Commons License.

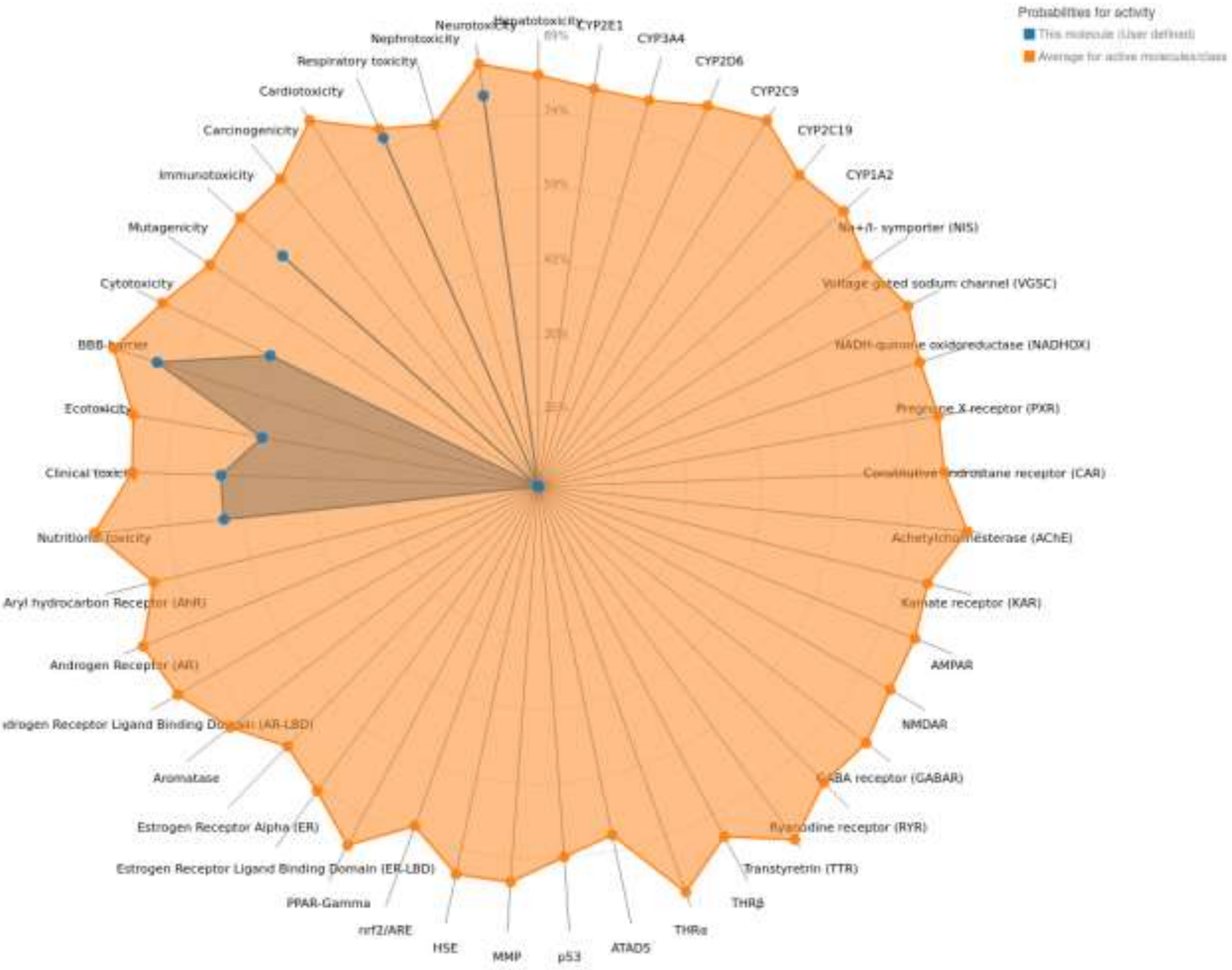
Probabilities for activity
This molecule (User defined)
Average for active molecules/class
Hepatotoxicity
Neurotoxicity
Nephrotoxicity
Respiratory toxicity
Cardiotoxicity
Carcinogenicity
Immunotoxicity
Mutagenicity
Cytotoxicity
BBB-barrier
Ecotoxicity
Clinical toxicity
Nutritional toxicity
Aryl hydrocarbon Receptor (AhR)
Androgen Receptor (AR)
Androgen Receptor Ligand Binding Domain (AR-LBD)
Aromatase
Estrogen Receptor Alpha (ER)
Estrogen Receptor Ligand Binding Domain (ER-LBD)
PPAR-Gamma
nrf2/ARE
HSE
MMP
p53
ATAD5
THRα
THRβ
Transtyretrin (TTR)
Ryanodine receptor (RYR)
GABA receptor (GABAR)
NMDAR
AMPAR
Kainate receptor (KAR)
Achetylcholinesterase (AChE)
Constitutive Androstane receptor (CAR)
Pregnane X receptor (PXR)
NADH-quinone oxidoreductase (NADHOX)
Voltage gated sodium channel (VGSC)
Na+/I- symporter (NIS)
CYP1A2
CYP2C19
CYP2C9
CYP2D6
CYP3A4
CYP2E1

Molecule 1

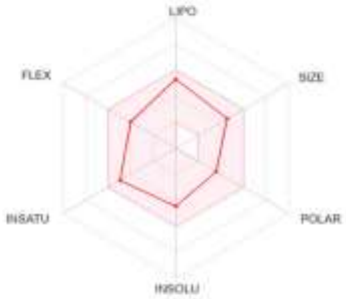

SMILES COc1cc(ccc1OC)C[C@H]1COC(=O)[C@@H]1Cc1ccc2c(c1)OCO2

Physicochemical Properties	
Formula	C21H22O6
Molecular weight	370.40 g/mol
Num. heavy atoms	27
Num. arom. heavy atoms	12
Fraction Csp3	0.38
Num. rotatable bonds	6
Num. H-bond acceptors	6
Num. H-bond donors	0
Molar Refractivity	98.15
TPSA	63.22 Å²

Lipophilicity	
Log $P_{o/w}$ (iLOGP)	3.28
Log $P_{o/w}$ (XLOGP3)	3.78
Log $P_{o/w}$ (WLOGP)	3.01
Log $P_{o/w}$ (MLOGP)	2.53
Log $P_{o/w}$ (SILICOS-IT)	4.28
Consensus Log $P_{o/w}$	3.37

Water Solubility	
Log *S* (ESOL)	-4.45
Solubility	1.31e-02 mg/ml ; 3.54e-05 mol/l
Class	Moderately soluble
Log *S* (Ali)	-4.80
Solubility	5.85e-03 mg/ml ; 1.58e-05 mol/l
Class	Moderately soluble
Log *S* (SILICOS-IT)	-5.96
Solubility	4.11e-04 mg/ml ; 1.11e-06 mol/l
Class	Moderately soluble

Pharmacokinetics	
GI absorption	High
BBB permeant	Yes
P-gp substrate	No
CYP1A2 inhibitor	No
CYP2C19 inhibitor	Yes
CYP2C9 inhibitor	Yes
CYP2D6 inhibitor	Yes
CYP3A4 inhibitor	Yes
Log K_p (skin permeation)	-5.88 cm/s

Druglikeness	
Lipinski	Yes; 0 violation
Ghose	Yes
Veber	Yes
Egan	Yes
Muegge	Yes
Bioavailability Score	0.55

Medicinal Chemistry	
PAINS	0 alert
Brenk	0 alert
Leadlikeness	No; 2 violations: MW>350, XLOGP3>3.5
Synthetic accessibility	3.72

Swiss Institute of Bioinformatics

SwissTargetPrediction

Home FAQ Help Download Contact Disclaimer

Query Molecule

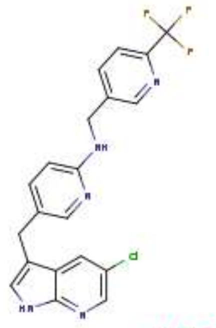

Target Classes

Top 15
Top 25
Top 50
All

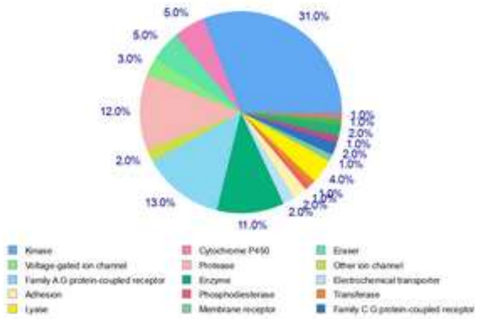

Export results:

Show 15 entries

Search:

Target	Common name	Uniprot ID	ChEMBL ID	Target Class	Probability*	Known actives (3D/2D)
Tyrosine-protein kinase receptor FLT3	FLT3	P36888	CHEMBL1974	Kinase		7 / 2
Cytochrome P450 2C9	CYP2C9	P11712	CHEMBL3397	Cytochrome P450		2 / 16
Cytochrome P450 2C19	CYP2C19	P33261	CHEMBL3622	Cytochrome P450		1 / 9
Cytochrome P450 3A4	CYP3A4	P08684	CHEMBL340	Cytochrome P450		2 / 29
Cytochrome P450 2D6	CYP2D6	P10635	CHEMBL289	Cytochrome P450		0 / 1
Cytochrome P450 1A2	CYP1A2	P05177	CHEMBL3356	Cytochrome P450		0 / 2
Histone deacetylase 3	HDAC3	O15379	CHEMBL1829	Eraser		22 / 0
Histone deacetylase 6	HDAC6	Q9UBN7	CHEMBL1865	Eraser		112 / 0
Histone deacetylase 4	HDAC4	P56524	CHEMBL3524	Eraser		155 / 0
Sodium channel protein type IX alpha subunit	SCN9A	Q15858	CHEMBL4296	Voltage-gated ion channel		45 / 0
Receptor-interacting serine/threonine-protein kinase 1	RIPK1	Q13546	CHEMBL5464	Kinase		16 / 0
Beta secretase 2	BACE2	Q9Y5Z0	CHEMBL2525	Protease		11 / 0
Insulin receptor	INSR	P06213	CHEMBL1981	Kinase		8 / 0
Tyrosine-protein kinase SRC	SRC	P12931	CHEMBL267	Kinase		18 / 0
Tyrosine-protein kinase HCK	HCK	P08631	CHEMBL3234	Kinase		7 / 0

Showing 1 to 15 of 100 entries

Previous 1 2 3 4 5 6 7 Next

*Probability for the query molecule - assumed as bioactive - to have this protein as target.
