# Supplementary material for: Role of CSF1R 550th-tryptophan in kusunokinin and CSF1R inhibitor binding and ligand-induced structural effect
Source: Sci Rep. 2024 May 31;14:12531. doi: 10.1038/s41598-024-63505-x (PMC11143223; doi:10.1038/s41598-024-63505-x)
Supplement: Supplementary file 1 — Supplementary Information. [file 41598_2024_63505_MOESM1_ESM.zip › Figure S1-Off-target-profiles-imatinib.pdf]

Figure S1. **Off-target profiles of imatinib**. All off-target predictions were performed using Protox 3.0 for cytotoxicity(https://tox.charite.de/protox3/), SwissADME for drug-likeness(http://www.swissadme.ch/), and SwissTargetPrediction for drug-target prediction (http://www.swisstargetprediction.ch/).

Toxicity Model Report

Copy Excel CSV PDF

Classification	Target	Shorthand	Prediction	Probability
Organ toxicity	Hepatotoxicity	dili	Active	0.71
Organ toxicity	Neurotoxicity	neuro	Active	0.92
Organ toxicity	Nephrotoxicity	nephro	Inactive	0.81
Organ toxicity	Respiratory toxicity	respi	Active	0.94
Organ toxicity	Cardiotoxicity	cardio	Inactive	0.84
Toxicity end points	Carcinogenicity	carcino	Inactive	0.67
Toxicity end points	Immunotoxicity	immuno	Active	0.66
Toxicity end points	Mutagenicity	mutagen	Inactive	0.73
Toxicity end points	Cytotoxicity	cyto	Inactive	0.52
Toxicity end points	BBB-barrier	bbb	Active	0.78
Toxicity end points	Ecotoxicity	eco	Active	0.65
Toxicity end points	Clinical toxicity	clinical	Active	0.61
Toxicity end points	Nutritional toxicity	nutri	Inactive	0.75

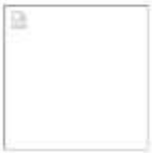

The toxicity radar chart is intended to quickly illustrate the confidence of positive toxicity results compared to the average of its class.
Click the thumbnail to access the plot once it has finished loading.

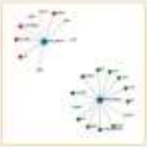

The network chart is intended to quickly illustrate the connection between the selected compound and predicted activities.
Click the thumbnail to access the plot once it has finished loading.

Oral toxicity prediction results for input compound

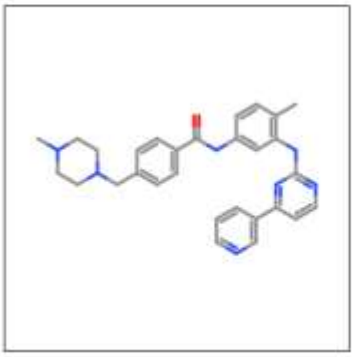

Predicted LD50: 100mg/kg

Predicted Toxicity Class: 3

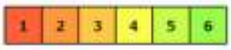

Average similarity: 48.13%

Prediction accuracy: 54.26%

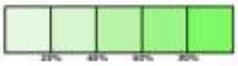

Name	
Molweight	493.6
Number of hydrogen bond acceptors	8
Number of hydrogen bond donors	2
Number of atoms	37
Number of bonds	41
Number of rotable bonds	8
Molecular refractivity	154.5
Topological Polar Surface Area	86.28
octanol/water partition coefficient(logP)	4.61

Print Toxicity Report

Comparison of input compound with dataset compounds

Value of input compound
Mean value of dataset

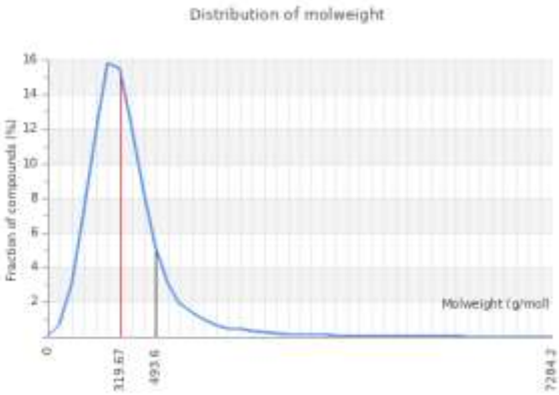

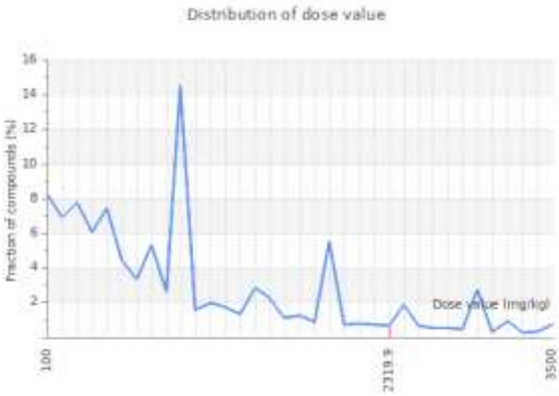

Toxicity targets

Possible binding to toxicity targets is shown below. For more information on the targets, please click on the individual abbreviations.

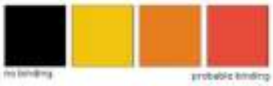

AA2AR	ADRB2	ANDR	AOFA	CRFR1	DRD3	ESR1	ESR2	GCR	HRH1	NR1I2	OPRK	OPRM	PDE4D	PGH1	PRGR

Last updated: March 2024
Disclaimer: Compound structures submitted will not be released under any circumstances. This work is licensed under a Creative Commons License.

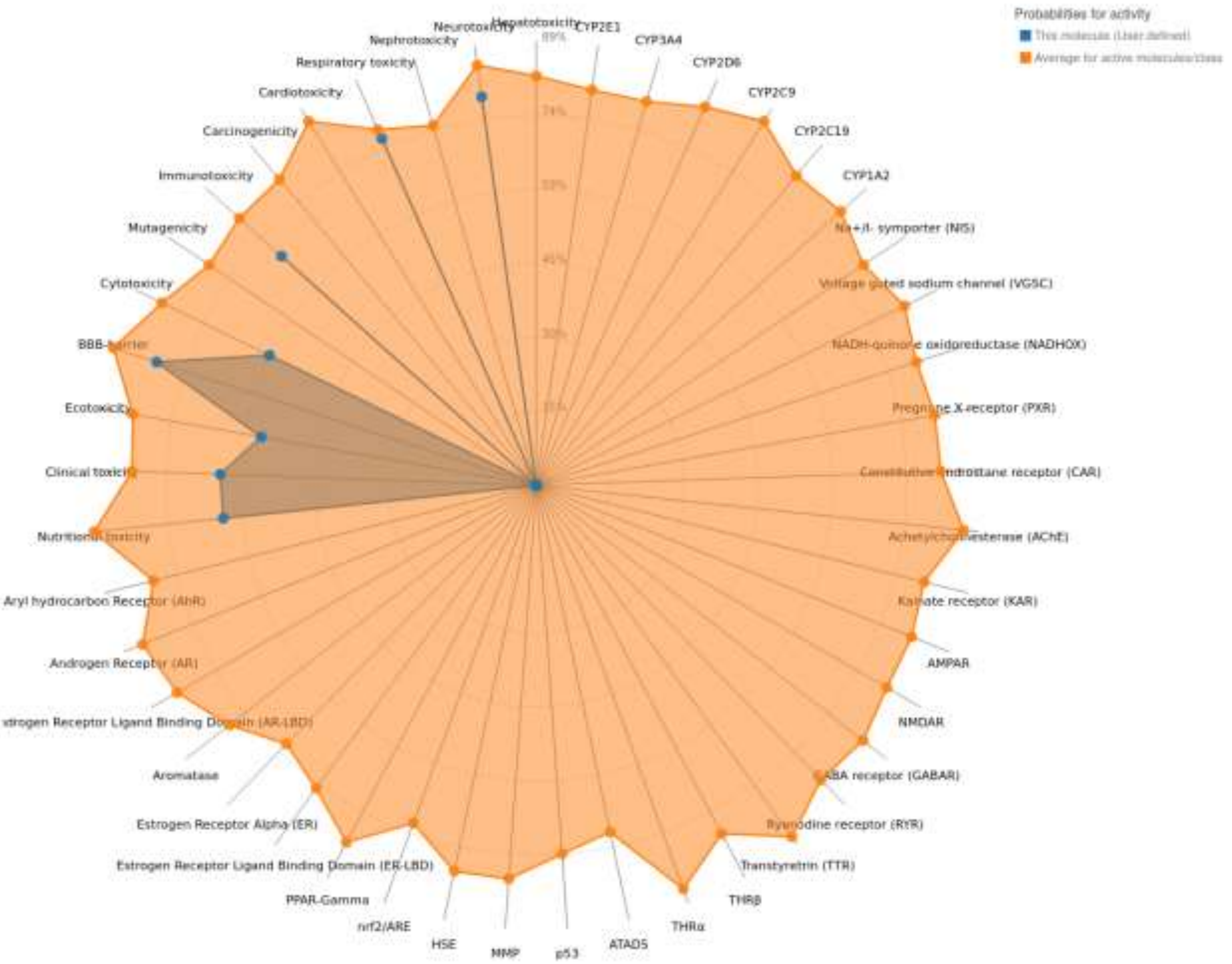
Probabilities for activity
This molecule (User defined)
Average for active molecules/class
Hepatotoxicity
Neurotoxicity
Nephrotoxicity
Respiratory toxicity
Cardiotoxicity
Carcinogenicity
Immunotoxicity
Mutagenicity
Cytotoxicity
BBB-barrier
Ecotoxicity
Clinical toxicity
Nutritional toxicity
Aryl hydrocarbon Receptor (AhR)
Androgen Receptor (AR)
drogen Receptor Ligand Binding Domain (AR-LBD)
Aromatase
Estrogen Receptor Alpha (ER)
Estrogen Receptor Ligand Binding Domain (ER-LBD)
PPAR-Gamma
nrf2/ARE
HSE
MMP
p53
ATAD5
THRα
THRβ
Transtyretrin (TTR)
Ryanodine receptor (RYR)
GABA receptor (GABAR)
NMDAR
AMPAR
Kainate receptor (KAR)
Achetylcholinesterase (AChE)
Constitutive Androstane receptor (CAR)
Pregnane X-receptor (PXR)
NADH-quinone oxidoreductase (NADHOX)
Voltage gated sodium channel (VGSC)
Na+/I- symporter (NIS)
CYP1A2
CYP2C19
CYP2C9
CYP2D6
CYP3A4
CYP2E1

Molecule 1

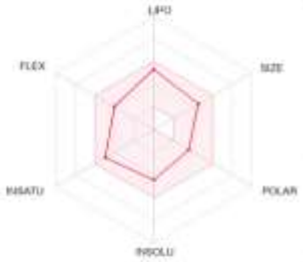

SMILES COc1cc(ccc1OC)C[C@H]1COC(=O)[C@@H]1Cc1ccc2c(c1)OCO2

Physicochemical Properties	
Formula	C21H22O6
Molecular weight	370.40 g/mol
Num. heavy atoms	27
Num. arom. heavy atoms	12
Fraction Csp3	0.38
Num. rotatable bonds	6
Num. H-bond acceptors	6
Num. H-bond donors	0
Molar Refractivity	98.15
TPSA	63.22 Å²

Lipophilicity	
Log $P_{o/w}$ (iLOGP)	3.28
Log $P_{o/w}$ (XLOGP3)	3.78
Log $P_{o/w}$ (WLOGP)	3.01
Log $P_{o/w}$ (MLOGP)	2.53
Log $P_{o/w}$ (SILICOS-IT)	4.28
Consensus Log $P_{o/w}$	3.37

Water Solubility	
Log *S* (ESOL)	-4.45
Solubility	1.31e-02 mg/ml ; 3.54e-05 mol/l
Class	Moderately soluble
Log *S* (Ali)	-4.80
Solubility	5.85e-03 mg/ml ; 1.58e-05 mol/l
Class	Moderately soluble
Log *S* (SILICOS-IT)	-5.96
Solubility	4.11e-04 mg/ml ; 1.11e-06 mol/l
Class	Moderately soluble

Pharmacokinetics	
GI absorption	High
BBB permeant	Yes
P-gp substrate	No
CYP1A2 inhibitor	No
CYP2C19 inhibitor	Yes
CYP2C9 inhibitor	Yes
CYP2D6 inhibitor	Yes
CYP3A4 inhibitor	Yes
Log K_p (skin permeation)	-5.88 cm/s

Druglikeness	
Lipinski	Yes; 0 violation
Ghose	Yes
Veber	Yes
Egan	Yes
Muegge	Yes
Bioavailability Score	0.55

Medicinal Chemistry	
PAINS	0 alert
Brenk	0 alert
Leadlikeness	No; 2 violations: MW>350, XLOGP3>3.5
Synthetic accessibility	3.72

Swiss Institute of Bioinformatics

SwissTargetPrediction

Query Molecule

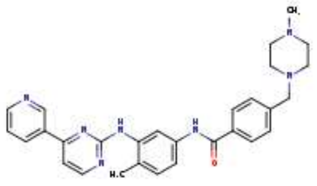

Target Classes

Top 15
Top 25
Top 50
All

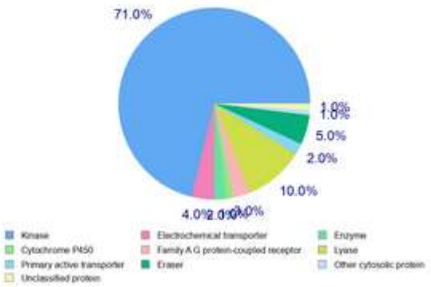

Export results:

Show 15 entries

Search:

Target	Common name	Uniprot ID	ChEMBL ID	Target Class	Probability*	Known actives (3D/2D)
Homeodomain-interacting protein kinase 4	HIPK4	Q8NE63	CHEMBL1075167	Kinase		15 / 1
Solute carrier family 22 member 2	SLC22A2	O15244	CHEMBL1743122	Electrochemical transporter		1 / 1
Multidrug and toxin extrusion protein 1	SLC47A1	Q96FL8	CHEMBL1743126	Electrochemical transporter		3 / 1
Multidrug and toxin extrusion protein 2	SLC47A2	Q86VL8	CHEMBL1743127	Electrochemical transporter		1 / 1
Phosphatidylinositol-5-phosphate 4-kinase type-2 gamma	PIP4K2C	Q8TBX8	CHEMBL1770034	Enzyme		3 / 2
Thromboxane-A synthase	TBXAS1	P24557	CHEMBL1835	Cytochrome P450		4 / 2
Tyrosine-protein kinase FYN	FYN	P06241	CHEMBL1841	Kinase		43 / 5
Macrophage colony stimulating factor receptor	CSF1R	P07333	CHEMBL1844	Kinase		243 / 14
Tyrosine-protein kinase ABL	ABL1	P00519	CHEMBL1862	Kinase		159 / 148
Serine/threonine-protein kinase RAF	RAF1	P04049	CHEMBL1906	Kinase		14 / 16
Platelet-derived growth factor receptor beta	PDGFRB	P09619	CHEMBL1913	Kinase		160 / 36
Stem cell growth factor receptor	KIT	P10721	CHEMBL1936	Kinase		140 / 56
Histamine H2 receptor	HRH2	P25021	CHEMBL1941	Family A G protein-coupled receptor		21 / 1
Tyrosine-protein kinase receptor FLT3	FLT3	P36888	CHEMBL1974	Kinase		243 / 106
Platelet-derived growth factor receptor alpha	PDGFRA	P16234	CHEMBL2007	Kinase		83 / 27

Showing 1 to 15 of 100 entries

Previous 1 2 3 4 5 6 7 Next
