# Supplementary figures and images for: Role of CSF1R 550th-tryptophan in kusunokinin and CSF1R inhibitor binding and ligand-induced structural effect
Source: Sci Rep. 2024 May 31;14:12531. doi: 10.1038/s41598-024-63505-x (PMC11143223; doi:10.1038/s41598-024-63505-x)

**Figure S5.** Molecular docking parameters. (A) Grid parameters. (B) Docking parameters.

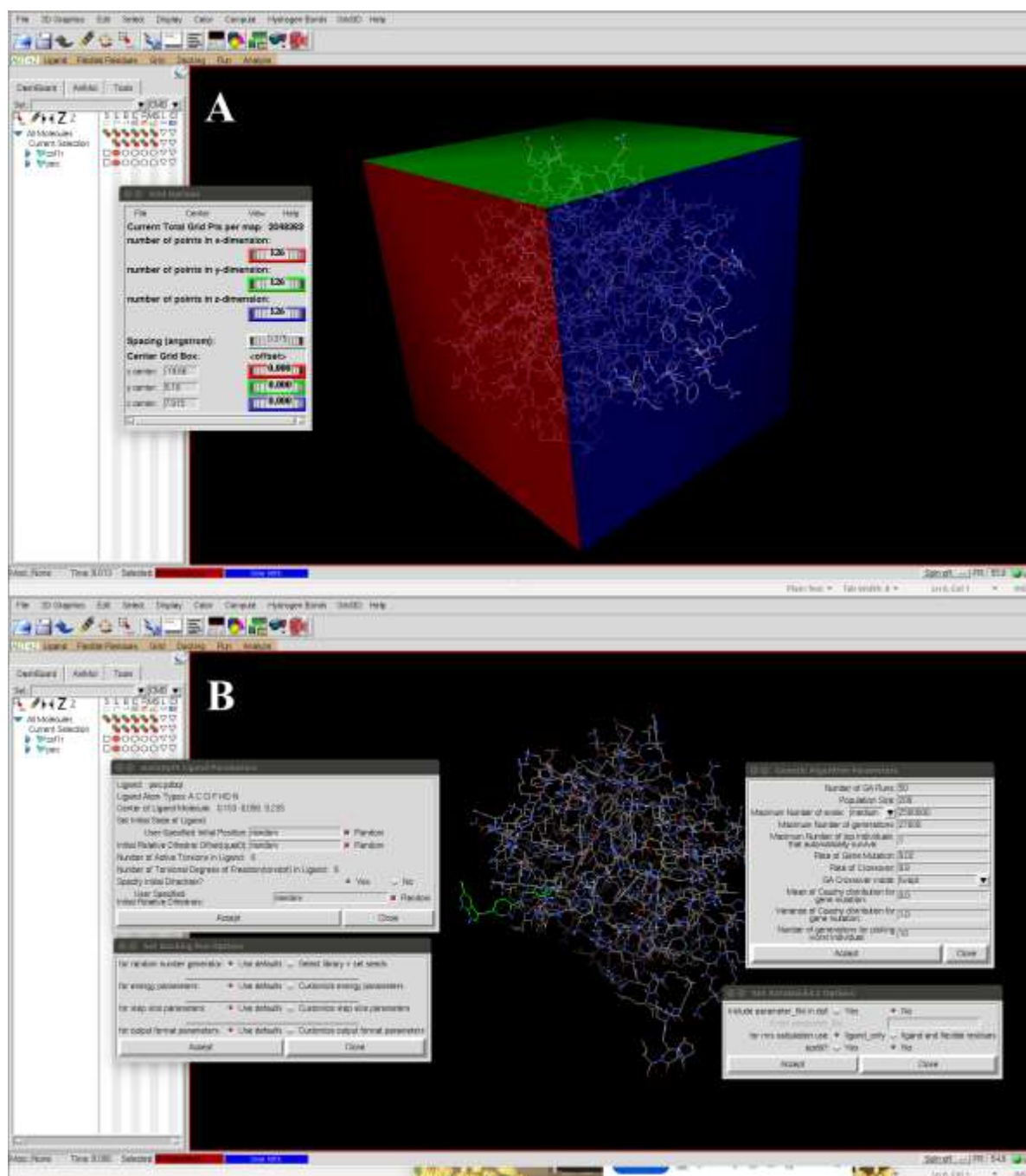

Supplement: Supplementary file 1 — Supplementary Information. [file 41598_2024_63505_MOESM1_ESM.zip › Figure-S5-Docking parameters.pdf]
